# Chemotaxonomic Classification of *Peucedanum japonicum* and Its Chemical Correlation with *Peucedanum praeruptorum*, *Angelica decursiva*, and *Saposhnikovia divaricata* by Liquid Chromatography Combined with Chemometrics

**DOI:** 10.3390/molecules27051675

**Published:** 2022-03-03

**Authors:** Jung-Hoon Kim, Eui-Jeong Doh, Guemsan Lee

**Affiliations:** 1 Division of Pharmacology, School of Korean Medicine, Pusan National University, Yangsan 50612, Korea; kmsct@pusan.ac.kr; 2 Research Center of Traditional Korean Medicine, Wonkwang University, Iksan 54538, Korea; bluemoon-lion@hanmail.net; 3 Department of Herbology, College of Korean Medicine, Wonkwang University, Iksan 54538, Korea

**Keywords:** *Peucedanum japonicum*, *Peucedanum praeruptorum*, *Angelica decursiva*, *Saposhnikovia divaricata*, genetic authentication, chemotaxonomic correlation

## Abstract

The roots of *Peucedanum japonicum* (Apiaceae) have been used as an alternative to the roots of *Saposhnikovia divaricata* (Apiaceae) to treat common cold-related symptoms in Korea. However, a variety of *Peucedanum* species, including the roots of *P. praeruptorum* or *Angelica decursiva* (=*P. decursivum*), have been used to treat phlegm–heat-induced symptoms in China. Hence, as there are differences in the medicinal application of *P. japonicum* roots between Korea and China, chemotaxonomic classification of *P. japonicum* was evaluated. Sixty samples derived from *P. japonicum*, *P. praeruptorum*, *A. decursiva*, and *S. divaricata* were phylogenetically identified using DNA barcoding tools, and chemotaxonomic correlations among the samples were evaluated using chromatographic profiling with chemometric analyses. *P. japonicum* samples were phylogenetically grouped into the same cluster as *P. praeruptorum* samples, followed by *S. divaricata* samples at the next cluster level, whereas *A. decursiva* samples were widely separated from the other species. Moreover, *P. japonicum* samples showed higher chemical correlations with *P. praeruptorum* samples or *A. decursiva* samples, but lower or negative chemical correlations with *S. divaricata* samples. These results demonstrate that *P. japonicum* is more genetically and chemically relevant to *P. praeruptorum* or *A. decursiva* and, accordingly, the medicinal application of *P. japonicum* might be closer to the therapeutic category of these two species than that of *S. divaricata*.

## 1. Introduction

Bang-Pung (Saposhnikoviae Radix), a traditional herbal medicine, has been used to treat common cold-induced disorders and relieve pain, and it originates from the roots of *Saposhnikovia divaricata* (Turcz.) Schischk. (Apiaceae) in Korean and Chinese pharmacopieas [1,2]. The roots of *Peucedanum japonicum* Thunb. (Apiaceae) are exclusively registered as “Sik-Bang-Pung” (Peucedani Japonici Radix; “Sik” means cultivated) in the Korean herbal pharmacopeia [3]. As the roots of *P. japonicum* are recognized as an alternative to Bang-Pung (the roots of *S. divaricata*), it is interesting that two different herbs belonging to two separate genera are used for the same medicinal purpose. Another notable issue is that the plant *P. japonicum* and its roots are botanically and medicinally named “Bin-Hae-Jeon-Ho” (bin hai qian hu; “Bin-Hae” means the plant lives near coastal areas), a type of “Jeon-Ho” in the Chinese literature [4,5]. Jeon-Ho, a medicinal name of Peucedani Radix, has been used to treat phlegm- and heat-induced respiratory disorders and it originates from the roots of *P. praeruptorum* Dunn (Baek-hwa-Jeon-Ho; “Baek-Hwa” means a white flower) or *Angelica decursiva* (Miq.) Franch. & Sav. (=*P. decursivum* (Miq.) Maxim.) (Ja-Hwa-Jeon-Ho; “Ja-Hwa” refer to a purple flower) [2,3]. Hence, whether the roots of *P. japonicum* can be used for different therapeutic purposes in Korea and China is controversial. This is because the roots of *P. japonicum* are used for treatment of common cold-related symptoms in Korea, while different *Peucedanum* species, including *P. japonicum*, are currently used to treat phlegm- and heat-induced respiratory symptoms in China [5]. Therefore, it is necessary to classify the roots of *P. japonicum* for medicinal purposes. Moreover, an investigation of the chemical relationship between *P. japonicum* and *S. divaricata*, *P. praeruptorum*, and *A. decursiva* can be crucial to estimate the therapeutic efficacy-based medicinal categorization of *P. japonicum*.

There are several types of adulterants still distributed in the market for various reasons, contributing to confusion owing to the differences in origin species depending on the country and morphological similarity, and misunderstandings also originate from common names of plants and their medicinal names. Various research methods have been applied to prevent this confusion. DNA-based genetic analysis is one such approach. This molecular biological research method targeting genomic DNA is not affected by the environment, and the reliability is high because of the clarity and reproducibility of the analyzed results [6].

In previous studies using DNA-based approaches for *S. divaricata*, *P. japonicum*, and *Glehnia littoralis*, restriction fragment length polymorphism analysis was performed using the international transcribed spacer (ITS) primer for genomic DNA [7], and random amplified polymorphic DNA (RAPD) analysis-based sequence characterized amplified region markers were developed [8]. DNA barcode sequence (ITS and chloroplast DNA region) analysis has also been reported [9]. For Peucedani Radix, several DNA-based approaches, RAPD analysis [10], and ITS nucleotide analysis [11,12] have already been performed. Recently, the chloroplast genome has been used to understand the identities, phylogeny, genetic populations, and evolution of plants using next-generation sequencing [13,14]. These results demonstrated that DNA-based gene differentiation is an efficient and accurate method for applications. Therefore, DNA barcode analysis, comprising ITS combined with four chloroplast DNA regions, was performed for the identification of samples used in this study.

Chemotaxonomic classification of the four aforementioned species using the chromatographic profiling method with chemometric analysis is a logical approach to investigate the chemical relationship between *P. japonicum* and the remaining three species. The chemical classification of *S. divaricata* and *P. japonicum* was performed by metabolic profiling analysis using ultra-performance liquid chromatography/quadrupole time-of-flight mass spectrometry and multivariate analysis [15]. *S. divaricata* root and its substitute, *P. ledebourielloides* root, have previously been distinguished using liquid chromatography/mass spectrometry-based metabolomics [16]. A high-performance liquid chromatography/photodiode array detector was used for the quantitative analysis of the marker compounds and the chemical differentiation between *S. divaricata* and *P. japonicum* roots and between *A. decursiva* and *P. praeruptorum* roots [17,18]. However, in contrast with diverse genetic studies, no studies have investigated the chemotaxonomic relationship between *P. japonicum*, *S. divaricata*, *P. praeruptorum*, and *A. decursiva.* Moreover, the *P. japonicum*, *S. divaricata*, *P. praeruptorum*, and *A. decursiva* samples used in the aforementioned articles were limited in their chemical analysis and were not apparently authenticated at the species level.

The genetic authentication-based chromatographic profiling method is an emerging technique that is mainly performed by DNA-barcoding hyphenated chromatographic analysis. The combined method provides species-level accuracy of herbal samples, and therefore, a more reliable classification of those samples can be acquired by chemical analysis [19]. This technique has been applied to the chemical classification of diverse herbal medicines, such as *Arnebia* [20], *Fritillaria* [21], *Phellodendron* [22], and *Daphne* [23] species. Our group has also previously developed a ‘genetic authentication-based chromatographic profiling’ method for chemically distinguishing between *Atractylodes* species and *Amomum* species, using ITS sequencing, HPLC analysis, and chemometrics [24,25,26].

In this research, we collected samples of the aforementioned four species and confirmed their genetic authentication using ITS sequence-based DNA barcoding analysis to establish the chemotaxonomic relevance of *P. japonicum* (PJ), *S. divaricata* (SD), *P. praeruptorum* (PP), and *A. decursiva* (AD). Thereafter, chemical profiling of the samples was carried out using an HPLC–diode array detector (HPLC–DAD) and chemotaxonomic classification was performed by chemometric analysis.

## 2. Results and Discussion

### 2.1. Internal Transcribed Spacer Regions of Nuclear Ribosomal Cistron

The nucleotide sequences of the ITS (ITS1, ITS2, include 5.8 s) were used for identification of the species of distributed Peucedani Radix, Peucedami Japonici radix and Saposhnikoviae Radix. Around 689–693 bp amplified nucleotide sequences were determined, based on the samples listed in Table 1 and Table 2. The determined sequences were confirmed using the Blast in NCBI GenBank data. In total, 44 site differences were observed among the four species shown in Table 1 and Table 2 (Table 3). A difference in 37 sites was observed between two species of Peucedani Radix (PJ and AD) with a 0.94 sequence identity matrix (Table S1). The two same genus samples, PP and PJ, had 16 site differences (sequence identity matrix 0.975; Table S1). SD and PJ had nine different sites (sequence identity matrix 0.986; Table S1). The results indicated that the ITS could be used as a clear and useful tool to identify the species of medicinal herbs in Table 1 and Table 2.

### 2.2. Chloroplast Genome-Based DNA Barcode Sequence Analysis

Four chloroplast DNA barcode regions (*rbcL*, *marK*, *psbA-trnH*, and *trnL-F* intergenic spacer) were analyzed to supplement the results of the ITS. The *psbA-trnH* region had the most variable sites among the four plastid barcode regions (except the ITS region). On the other hand, the *trnL-F* intergenic spacer was the most conserved region among all of the analyzed DNA barcode regions (including ITS).

Although four analyzed plastid loci had less abundant variable sites and were highly conserved compared to the ITS region, it could be useful to separate the species used in this study.

To obtain more detail, a 390F/1326R primer set was used to amplify the *matK*, and the 933-base partial nucleotide sequences were determined. The total number of variable sites was 13; however, some of them were highly conserved. In particular, there were only two site differences between PP and PJ. Nevertheless, *matK* could distinguish four species sufficiently.

For the *rbcL* with a *rbcL* a-f/724R primer set, 670-base partial nucleotide sequences were determined in all samples listed in Table 1 and Table 2. Six variable sites were found, which was a well-conserved region among the five DNA barcode regions. The sequence identity matrix of *rbcL* was 0.992–1.000. In the *rbcL* region, PP and PJ showed identical nucleotide sequences (sequence identity matrix 1, Supplementary Materials Table S1). Except for this case, other species could be identified by comparing *rbcL* nucleotide sequences.

The *psbA-trnH* with a *trnH2*/*psbAF* primer set showed different lengths of amplified product dependent on the species, and around 311–345 base partial nucleotide sequences were determined. The *psbA-trnH* had the shortest aligned length among the five DNA barcode regions but showed the most variable sites (17) among the four chloroplast barcode regions, and had several indel sequences. Therefore, the sequence identity matrix ranged from 0.904 to 0.944. Between PP and PJ, the closest result was 0.944.

A *trnL-e/trnL-f* primer set was used for the *trnL-F* intergenic spacer, and 444-base nucleotide sequences were identified. As mentioned earlier, the *trnL-F* intergenic spacer was the most conserved region, and the sequence identity matrix ranged from 0.99 to 1.00. Four variable sites were observed in AD alone, and the other three species showed the same sequence.

A single region chloroplast DNA barcode analysis approach and be inaccurate in determining the species’ identity. Therefore, the Consortium for the Barcode of Life (CBOL)-Plant working group also recommends two- or more locus combinations to initiate the barcode process for plant species. The recommended standard combination locus is *rbcL-matK*, because this combination is a practical solution to the complex balance between universality, sequence quality classification, and cost. [27]. However, this combination is not always efficient and, in this case, three- or more locus analyses are necessary. Therefore, the *psbA-trnH* and *trnL-F* intergenic spacer regions were additionally selected for analysis in this study. Both loci were quite short, which suggests that they could practically be used for the analysis of processed distributed samples. Unfortunately, the *trnL-F* intergenic spacer regions of the four species shown in Table 1 and Table 2 were highly conserved. In contrast, the *psbA-trnH* is already known as one of the most variable genome segments in the angiosperm chloroplasts. As expected, it had most variable sites in this study, but it also had many indel sequences. Therefore, single-locus analysis was not efficient.

### 2.3. Phylogenetic Analysis

**Figure 1:**
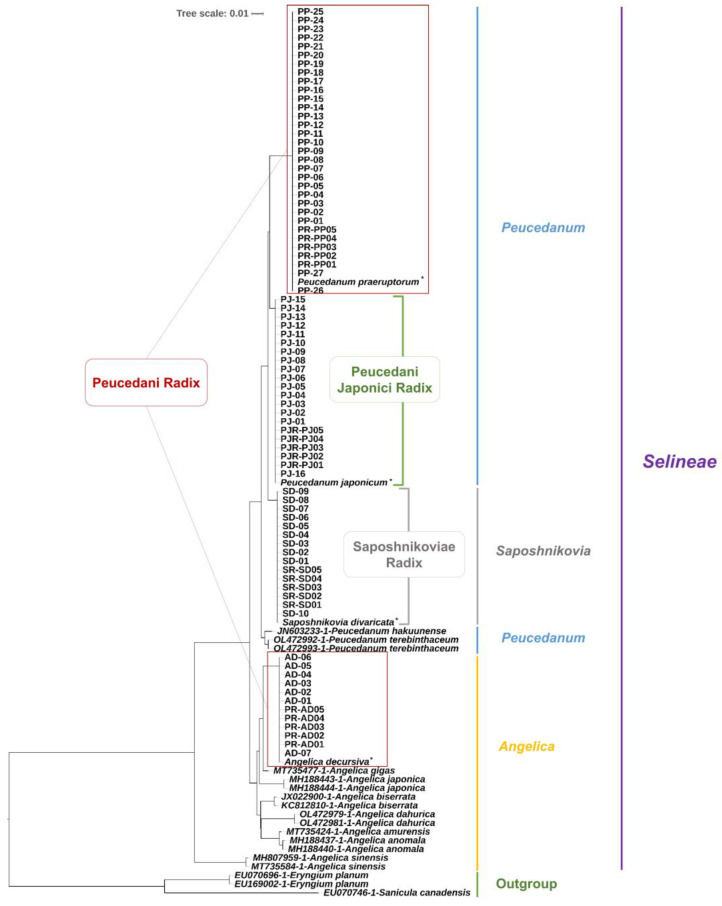
Maximum likelihood-based inference of phylogenetic tree with Smart Model Selection, constructed based on the combined five DNA barcode regions (ITS and four plastids). PJ: *Peucedanum japonicum*, PP: *P. praeruptorum*, AD: *Angelica decursiva*, SD: *Saposhnikovia divaricata.* ‘*’ represents the NCBI Genbank data combination.

To analyze the genetic relationship among the four species used in this study, the PhyML + SMS (Maximum likelihood-based inference of phylogenetic trees with Smart Model Selection) program was performed with concatenated nucleotide sequences of ITS and four chloroplast DNA barcodes. Four species commonly located in the Selineae tribe of the Apiacae family are clustered into two groups, genus Angelica and genus *Peucedanum* (including genus *Saposhnikovia*) (Figure 1). The results of genetic flexibility analysis of the four species show that PP and PJ are very close. Comparing the results with the plants of genus *Peucedanum*, PP and PJ, shows that they are not only the same genus, but genetically closer to other species. AD is located in the genus *Angelica* group and is relatively far in genetic distance compared to the other three species.

### 2.4. Chromatographic Profiling of PJ, PP, AD, and SD Samples

HPLC analytical conditions, including the mobile phase modifier, mobile phase composition, and UV detection wavelength, were optimized for the chromatographic analysis of the samples. The addition of 0.1% trifluoroacetic acid (TFA; *v*/*v*) in water with acetonitrile was chosen owing to better inter-peak separation and clearer detection of peaks under the acidic mobile phase in the mobile phase without TFA. The UV detection wavelengths were selected based on the optimal absorbance of each peak as follows: 4 peaks at UV 235 nm, 13 peaks at UV 250 nm, 14 peaks at UV 275 nm, 4 peaks at UV 300 nm, 10 peaks at UV 310 nm, 46 peaks at UV 325 nm, 7 peaks at UV 323 nm, and 2 peaks at UV 350 nm (Supplementary Materials Table S2).

The intraday precision of the sample was <0.2% for retention time and <4.0% for the absolute peak area, and interday precision was <0.1% for retention time and <3.0% for the peak area (Supplementary Materials Table S3).

**Figure 2:**
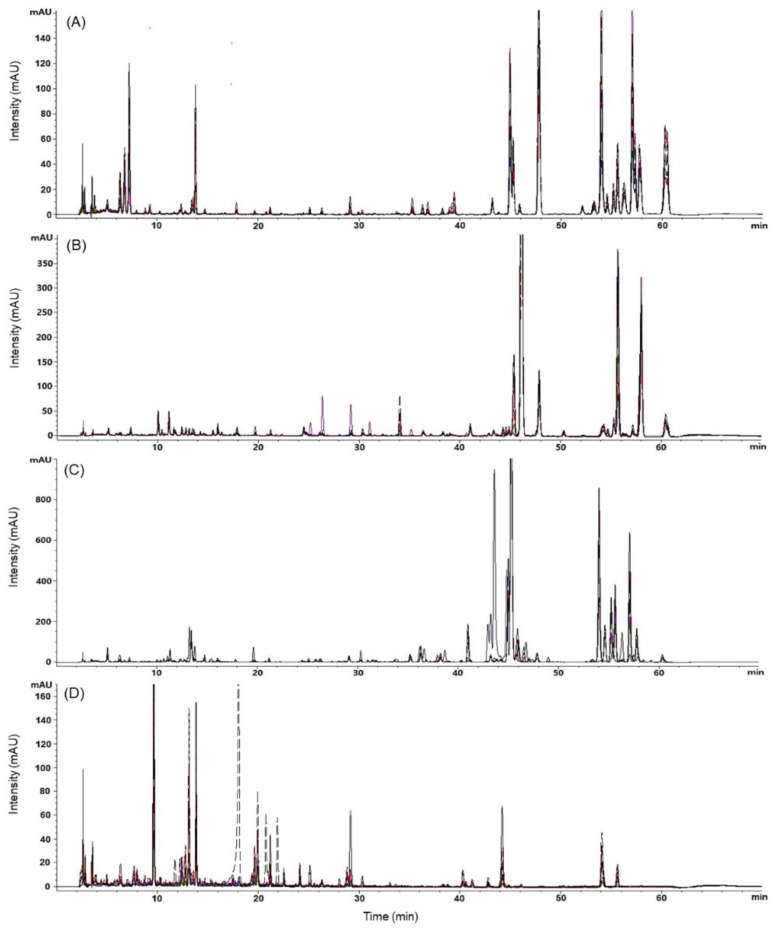
Overlapping chromatograms of representative samples of *Peucedanum japonicum* ((**A**) PJ01–10), *P. praeruptorum* ((**B**) PP01–10), *Angelica decursiva* ((**C**) AD01–07), and *Saposhnikovia divaricata* ((**D**) SD01–10) at a detection wavelength of 325 nm.

The overlapping intra-species chromatograms showed similar patterns within the samples of each species, except for a few outliers in the AD and SD samples, whereas those of inter-species comparisons showed slightly different patterns among PJ, PP, and AD samples, with a few identical peaks for PJ and AD samples after a retention time of 40 min. The chromatographic patterns of the SD samples were distinguishable from those of the PJ, PP, and AD samples over the entire retention time (Figure 2 and Figure S1).

Among the peaks commonly occurring in more than two species, the average peak areas of many peaks were significantly different between two species as follows: 26 peaks between PJ and AD samples (peaks 1, 3, 4, 14, 17, 18, 23, 33, 46, 47, 49, 52, 53, 54, 64, 69, 70, 76, 88, 90, 91, 92, 95, 97, 99, and 100); 15 peaks between PJ and PP samples (peaks 2, 4, 6, 14, 17, 18, 23, 39, 70, 76, 88, 92, 93, 95, and 100); 4 peaks between PJ and SD samples (peaks 2, 18, 36, and 45); 27 peaks between PP and AD samples (peaks 1, 6, 11, 12, 13, 17, 23, 39, 49, 53, 61, 64, 70, 76, 79, 81, 82, 83, 84, 85, 87, 88, 90, 91, 93, 94, 95, and 100); 17 peaks between PP and SD samples (peaks 6, 12, 15, 17, 18, 22, 36, 45, 57, 61, 62, 66, 73, 81, 83, 85, and 92); 10 peaks between AD and SD samples (peaks 18, 45, 56, 79, 81, 82, 83, 84, 85, and 92) (Figure S2).

### 2.5. Clustering Analysis of the Samples Using Chemometric Statistical Methods

**Figure 3:**
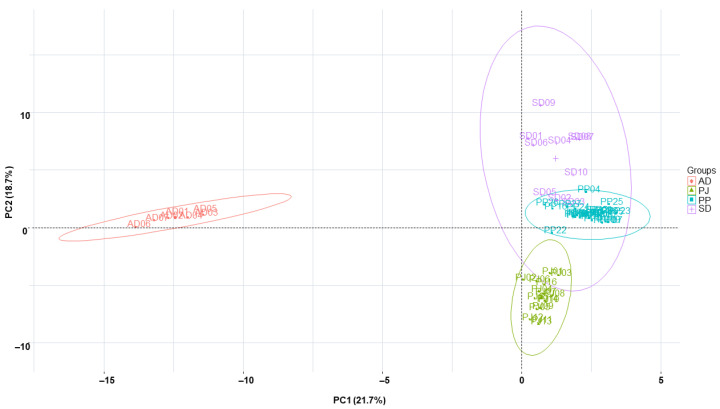
Score plot of principal components (PC1 vs. PC2) based on the variables (absolute area of reference peaks) with *Peucedanum japonica*, *P. praeruptorum*, *Angelica decursiva*, and *Saposhnikovia divaricata* samples based on a 99% confidence ellipse. PC1 and PC2 represent 21.7% and 18.7% of the total variance, respectively. PJ: *Peucedanum japonicum*, PP: *P. praeruptorum*, AD: *Angelica decursiva*, SD: *Saposhnikovia divaricata*.

The chemotaxonomic correlations between PJ samples and PP, AD, and SD samples were measured using chemometric clustering tools, principal component analysis, and *k*-means clustering analysis. As shown in Figure 3, the samples of each species formed distinct clusters based on their own origins in the principal component (PC) scores, PC1 and PC2, which explained 21.7% and 18.7% of the total variance, respectively. PJ, PP, and AD samples were densely located in their own species groups which were clearly separated from each other. Although the SD samples also formed clusters, they were distributed widely in the SD group, resulting in the interruption of a few samples (SD02 and SD03) in the PP group. The PJ samples were located closer to the PP samples than to the SD samples, whereas the AD samples were exceptionally different from the other sample groups in terms of PC1 and PC2 scores.

**Figure 4:**
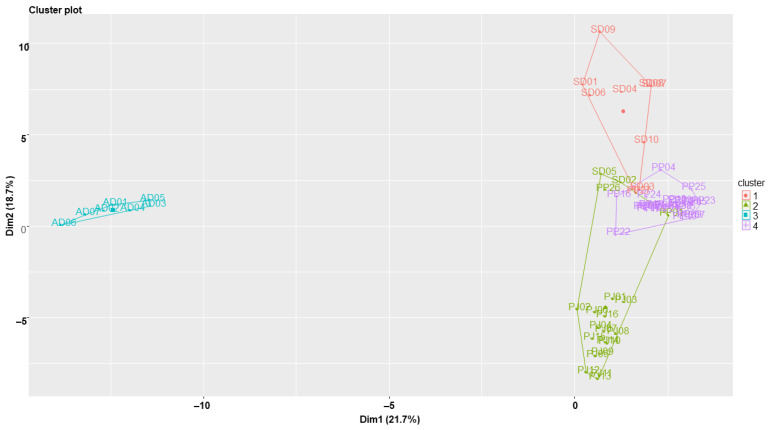
*k*-Means clustering plot of *Peucedanum japonica*, *P. praeruptorum*, *Angelica decursiva*, and *Saposhnikovia divaricata* samples. Dim: dimension. PJ: *Peucedanum japonicum*, PP: *P. praeruptorum*, AD: *Angelica decursiva*, SD: *Saposhnikovia divaricata*.

In the *k*-means clustering analysis, four clusters were selected as the optimal number of clusters, which was determined using the silhouette method (Figure S3). The distribution of samples according to their dimension scores was consistent with those in the PC plot. However, the grouping of samples was different, as follows: PP01, PP26, SD02, and SD05 samples were contained in the PJ cluster and PP27 was contained in the SD cluster. Hence, the PJ cluster was intruded by the PP cluster which also slightly overlapped with the SD cluster. The AD cluster also showed a distinct distance from the other clusters, as shown in the PC plot (Figure 4).

The results of clustering analyses demonstrate that the PJ samples showed better chemical proximity with the PP samples than the SD samples due to the PC (=dimension score in *k*-means) scores of the PJ samples being closer to those of the PP samples than the SD samples [28,29].

### 2.6. Evaluation of Similarity between the Samples Using Pearson’s Correlation Coefficient

**Figure 5:**
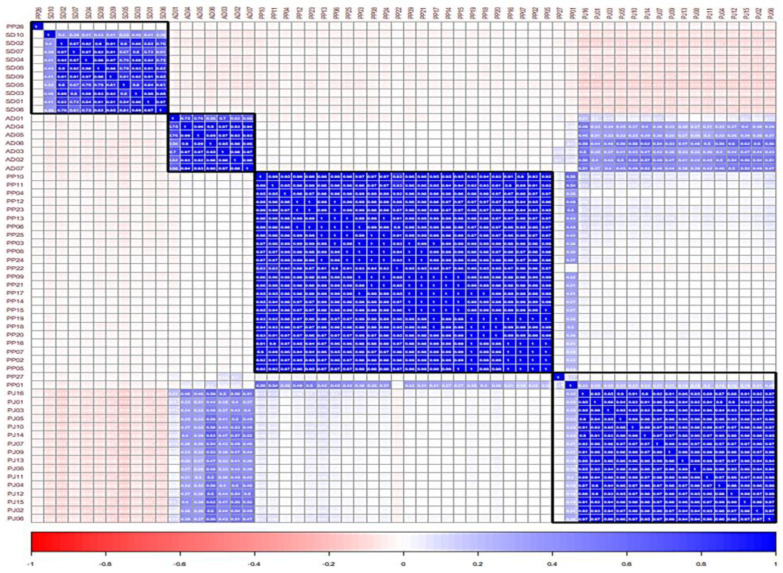
Correlation plot of the Pearson’s correlation coefficient (*r*) among *Peucedanum japonica*, *P. praeruptorum*, *Angelica decursiva*, and *Saposhnikovia divaricata* samples. The range of the coefficient is –1 (red color) < *r* < 1 (blue color). PJ: *Peucedanum japonicum*, PP: *P. praeruptorum*, AD: *Angelica decursiva*, SD: *Saposhnikovia divaricata*.

The similarity among the PJ, PP, AD, and SD samples was evaluated using Pearson’s correlation coefficient (*r*), which ranged from −1 to +1 (Table S4). In the correlation plot, the PJ samples showed a higher correlation with the AD samples (*r* = 0.791 for mean and 0.976 for median), followed by most PP samples (*r* = 0.029 for mean and 0.021 for median), and were negatively correlated with the SD samples (*r* = −0.099 for mean and −0.097 for median). The correlations between the PP and AD samples (*r* = −0.011 for mean and −0.014 for median), PP and SD samples (*r* = −0.038 for mean and median), and AD and SD samples (*r* = −0.061 for mean and −0.060 for median) were not different. The mean and median *r* values also showed that the intra-species relationships were as follows: PJ–PJ > PP–PP = AD–AD > SD–SD. Meanwhile, inter-species relationships were as follows: PJ–AD > PJ–PP > PP–AD > PP–SD > AD × SD > PJ–SD (Figure 5 and Table 4).

The average *r* values of the samples of each species relative to the individual samples of other independent species exhibited diverse intra- and inter-sample variations compared to the rest of the species samples, that is, higher correlations for PP01–PJ samples, PP01– and PP27–AD samples, and PP26–SD samples and lower correlation for AD01–PJ samples, PP01–PP samples, AD01–PP samples, AD01–AD samples, and SD10–SD samples (Figures S4–S7).

Positive and higher coefficient (*r*) values between the PJ and the AD samples indicate a stronger inter-species correlation, as compared with the weaker inter-species correlation between the PJ and the PP samples, with lower *r* values. Negative and weaker *r* values between the PJ and the SD samples indicated that their inter-species similarity was lower than those with positive *r* values [30,31].

The chemotaxonomic classification of PJ, PP, AD, and SD samples based on phylogenetic authentication was successfully evaluated using chromatographic profiling combined with chemometric analysis, and their chemical characteristics were clearly reflected by their own species. The chemical correlation between PJ and the other three species was also investigated, and it was observed that PJ had a strong chemical relationship with PP and AD, depending on the measured chemometric analyses; however, its chemical relationship with SD was less or even negligible.

As the therapeutic activity of herbal medicines can possibly be explained by their chemical constituents, the chemotaxonomic closeness between PJ and PP or AD might provide evidence for their analogous medicinal application to reduce phlegm and heat-induced respiratory symptoms. Meanwhile, the chemotaxonomic distance between PJ and SD presumably indicates their therapeutic dissimilarity with respect to relieving common cold-induced disorders. Further pharmacological and clinical studies are required to confirm these chemotaxonomic results.

Nonetheless, there are a few limitations to this study, as follows: (1) sample numbers for AD or SD were insufficient, mainly owing to difficulties in collecting genuine species; (2) intra-species chemical variation in SD samples was prominent, presumably owing to diverse habitats [32,33], and this could be caused by environmental factors, including temperature, dryness/humidity, rainfall, soil conditions, and altitude, which definitely affect the production of secondary metabolites in herbal plants [34,35,36]; (3) insufficient research on chemical classifications among the aforementioned four species.

## 3. Materials and Methods

### 3.1. Plant Materials and Reagents

Methanol, water, and acetonitrile (HPLC grade) were purchased from J. T. Baker (Phillipsburg, NJ, USA). TFA was purchased from Sigma-Aldrich (St. Louis, MO, USA). Prim-O-glucosyl-cimifugin (peak 10), cimifugin (peak 19), ubelliferone (peak 21), sec-O-glucosyl-hamaudol (peak 34), psoralen (peak 42), xanthotoxin (peak 44), bergapten (peak 45), oxypeucedanin (peak 48), imperatorin (peak 57), decursin (peak 66), praeruptorin A (peak 73), praeruptorin B (peak 92), and praeruptorin C (peak 98) were purchased from ChemFaces (Wuhan, China).

Sixteen samples of PJ, twenty-seven samples of PP, seven samples of AD, and ten samples of SD were collected from agricultural plantations, natural habitats, and markets in Korea and China, and were also supplied by the Korea Institute of Oriental Medicine (Table 1). The collected samples were morphologically authenticated by the authors (J.H. Kim and G. Lee). For genetic identification, 20 additional voucher samples were used as standard reference samples (Table 2). All voucher specimens (code No. PNUKM-2021-PJ01–PJ16, PP01–PP27, AD01–AD07, and SD01–SD10) and extracted genomic DNA were deposited at the herbarium of the College of Korean Medicine in Wonkwang University and at the School of Korean Medicine, Pusan National University.

### 3.2. Preparation of Genomic DNA

A NucleoSpin^®^ Plant II kit (Macherey-Nagel, Dueren, Germany) with PL1 lysis buffer was used to extract the genomic DNA of the samples in Table 1 and Table 2. Some samples required extra steps to treat 10% cetyltrimethylammonium bromide with 0.7 M NaCl added to remove high levels of phenolic compounds and/or polysaccharides.

### 3.3. PCR Amplification for DNA Barcode Analysis

For DNA barcode analysis, PCR amplification was performed using a T-personal cycler (Biometra, Jena, Germany). Briefly, 1X AccuPower^®^ GoldHotStart Taq PCR PreMix (Bioneer, Daejeon, Korea) with 600 nM primer set and 30 ng of genomic DNA were used for each PCR amplification. The primer set ITS1 (5′-TCCGTAGGTGAACCTGCGG-3′) and ITS4 (5′-TCCTCCGCTTATTGATATGC-3′) [37] were used to amplify the ITS include 5.8 s. For the chloroplast DNA barcodes, *rbcL* a–f (5′-ATGTCACCACAAACAGAGACTAAAGC-3′) /724R (5′-TCGCATGTACCTGCAGTAGC-3′) and 390F (5′-CGATCTATTCA TTCAATATTTC-3′) /1326R (5′-TCTAGCACACGAAAAGTCGAAGT-3′) primer sets were used for *rbcL* and *matK* amplification, respectively [38,39,40]. For the *trnL-F* intergenic spacer, the *trnL-e* (5′-GGTTCAAGTCCCTCTTATCCC-3′)/*trnL-f* (5′-ATTTGAACTGGTGACACGAG-3′) set and the primer set *trnH2* (5’-CGCGCATGGTGGATTCACAATCC-3’)/*psbAF* (5’-GTTATGCATGAACGTAATGCTC-3’) were used for the *psbA-trnH* regions [27,41]. The amplified products were separated and confirmed by electrophoresis by using 1.5% agarose gel added with Safe-View^TM^ (abm, Richmond, VA, Canada).

### 3.4. Determination of DNA Sequences of PCR Product

All of the PCR products were sub-cloned after being separated from agarose gels by use of a TOPcloner™ TA Kit (Enzynomics, Daejeon, Korea). The DNA sequences were then determined through an interpretation performed by Bioneer (Daejeon, Korea). To improve the accuracy of the results, the DNA barcode analysis was repeated three times, independently from the genomic DNA extraction stage.

### 3.5. Analysis of DNA Sequences and Preparation of Dendrogram

The determined DNA sequences were first analyzed using Bioedit’s ClustalW multiple sequence alignment (Bioedit, v7.0.9; available from http://www.mbio.ncsu.edu/BioEdit/page2.html, accessed on 28 November 2021) and reconfirmed with multiple sequence alignment using MAFFT (MAFFT, v7; available from https://mafft.cbrc.jp/alignment/server/, accessed on 29 November 2021) [42]. To confirm the polymorphism represented by IUPAC symbols, all sequences were identified at least twice using a chromatogram of nucleotide sequences provided by the Bioneer sequencing service. The phylogenetic tree for the ITS region was analyzed using MAFFT (multiple alignment, v7.407_1), BMGE (alignment curation, v.1.12_1) [43] and PhyML (tree inference based on the maximum-likelihood, v.3.1_1) [44,45] workflow (PhyML/OneClick, available from https://ngphylogeny.fr/, accessed on 3 December 2021). Phylogenetic analysis of the combined five DNA barcodes (ITS and four plastid regions) was followed by the PhyML+SMS/OneClick method, which was performed in accordance with MAFFT, BMGE, and PhyML+SMS (maximum likelihood-based inference of phylogenetic trees with Smart Model Selection, available from https://ngphylogeny.fr/, accessed on 8 December 2021) [46]. All sequence results completed in the analysis were compared and confirmed with the NCBI GenBank using Blast [47]. The reference data ITS nucleotide sequences for analysis of the genetic relationships comes from NCBI Genbank and were represented by the accession number in phylogenetic tree. The Chloroplast barcode data were also collected from NCBI Genbank, including MT671397.1, NC033344.1, OL362112.1, MW436378.1, MW820164.1, MW900177.1, MT921980.1, MT921.997, and KX352468.1. Two species, *Eryngium planum* (EU169002, EU0706969, MT561039.1) and *Sanicula canadensis* (EU070746.1, KP642834.1, KP643255.1, KJ773865.1), were used as the outgroup [48].

### 3.6. Preparation of Samples for HPLC Analysis

The pulverized PJ, SD, PP, and AD samples were homogenized using a 500 μm testing sieve (Chunggyesanggong-sa; Gunpo, Gyeonggi, Korea). The extraction was performed using a powder of 500 mg with 5 mL of methanol using an ultrasonic extractor (Power Sonic 520; Hwashin Tech, Daegu, Korea) for 30 min. The extract, filtered through a 0.2 μm syringe filter (BioFact, Daejeon, Korea), was evaporated using a nitrogen-blowing concentrator (MGS2200; Eyela, Miyagi, Japan) and was re-dissolved in HPLC-grade methanol at a concentration of 20,000 μg/mL prior to HPLC injection.

### 3.7. HPLC Conditions for Chromatographic Profiling

Chromatographic analysis was performed using an Agilent 1200 liquid chromatography system (Agilent Technologies; Palo Alto, CA, USA) consisting of an autosampler, degasser, solvent pump, and DAD. Data were processed using ChemStation (Agilent Technologies). The chemical constituents, including 13 marker compounds, were separated on a Capcell Pak Mg II C_18_ column (4.6 mm × 250 mm, 5 μm; Shiseido, Tokyo, Japan) at 35 °C with 1 mL/min flow rate and 10 μL injection volume. The mobile phase consisted of water containing 0.1% TFA (A) and acetonitrile (B), with the following gradient elution: 15% (B) over 0–2 min, 15–50% (B) over 2–30 min, 50% (B) over 30–32 min, 50–75% (B) over 32–55 min, 75% (B) over 55–58 min, and then re-equilibrated to 15% (B) until the end of the analysis. Detection was performed using a UV detector at wavelengths of 235, 250, 275, 300, 310, 325, 335, and 350 nm.

The precision of the analytical methods was determined by analyzing the retention times and absolute areas of selected peaks of PJ samples within one day (intraday precision) and over three consecutive days (interday precision). Precision was represented as relative standard deviations (RSDs), where RSD (%) = ((standard deviation/mean value) × 100).

### 3.8. Chemometric Statistical Analysis

The genetically identified samples of PJ, PP, AD, and SD were analyzed using HPLC, and the chemical relationship between the samples was evaluated using chemometric tools, that is, principal component analysis, *k*-means cluster analysis, and Pearson’s correlation analysis. In total, 100 peaks were selected as profiling peaks, which were >1.0% of the total peak area. Sixty samples and the absolute area of each profiling peak were used as a matrix for construction of the PC plot, the *k*-means cluster plot, and for the calculation of Pearson’s correlation coefficient. The difference between the absolute area of each peak from the samples of independent species was evaluated using Tukey’s test, with significance at *p* < 0.05, *p* < 0.01, and *p* < 0.001. Chemometric analyses and Tukey’s test were conducted using the open source software R (v. 4.1.2; The R Foundation for Statistical Computing).

## 4. Conclusions

Overall, 60 samples of PJ, PP, AD, and SD were phylogenetically authenticated using ITS and chloroplast genome-based DNA barcoding analysis at the species level. Chemotaxonomic classification of PJ and its chemical correlation with the remaining three species were investigated using chromatographic profiling with chemometric analyses. PJ samples, which showed the closest phylogenetic relationship with PP samples, showed a stronger chemical correlation with PP or AD samples but a weaker or even negative chemical correlation with SD samples. The results from the phylogenetic analysis-hyphenated chemotaxonomic correlation suggested the transfer of PJ from SD to the category of PP or AD for medicinal applications. Further pharmacological and clinical study would be necessary to support the chemical re-categorization of PJ.

## Figures and Tables

**Table 1:** Species identification of the samples using the combined five DNA barcode analysis.

Code	Species Identification	Geographic Origin	Code	Species Identification	Geographic Origin
PJ-01	*Peucedanum japonicum*	-	PP-15	*P. praeruptorum*	China
PJ-02	*P. japonicum*	Yeongcheon, Gyeongbuk, Korea	PP-16	*P. praeruptorum*	Zhejiang, China
PJ-03	*P. japonicum*	-	PP-17	*P. praeruptorum*	Zhejiang, China
PJ-04	*P. japonicum*	Yeongju, Gyeongbuk, Korea	PP-18	*P. praeruptorum*	Zhejiang, China
PJ-05	*P. japonicum*	-	PP-19	*P. praeruptorum*	Zhejiang, China
PJ-06	*P. japonicum*	Gyeongbuk, Korea	PP-20	*P. praeruptorum*	Zhejiang, China
PJ-07	*P. japonicum*	Gyeongbuk, Korea	PP-21	*P. praeruptorum*	China
PJ-08	*P. japonicum*	Yeongju, Gyeongbuk, Korea	PP-22	*P. praeruptorum*	-
PJ-09	*P. japonicum*	Gyeongbuk, Korea	PP-23	*P. praeruptorum*	-
PJ-10	*P. japonicum*	Yeongju, Gyeongbuk, Korea	PP-24	*P. praeruptorum*	-
PJ-11	*P. japonicum*	Korea	PP-25	*P. praeruptorum*	China
PJ-12	*P. japonicum*	Hwasun, Jeonnam, Korea	PP-26	*P. praeruptorum*	Yunnan, China
PJ-13	*P. japonicum*	Bonghwa, Gyeongbuk, Korea	PP-27	*P. praeruptorum*	Zhejiang, China
PJ-14	*P. japonicum*	Yeongju, Gyeongbuk, Korea	AD-01	*Angelica decursiva*	-
PJ-15	*P. japonicum*	Bonghwa, Gyeongbuk, Korea	AD-02	*A. decursiva*	-
PJ-16	*P. japonicum*	Korea	AD-03	*A. decursiva*	-
PP-01	*P. praeruptorum*	China	AD-04	*A. decursiva*	-
PP-02	*P. praeruptorum*	China	AD-05	*A. decursiva*	-
PP-03	*P. praeruptorum*	China	AD-06	*A. decursiva*	-
PP-04	*P. praeruptorum*	China	AD-07	*A. decursiva*	China
PP-05	*P. praeruptorum*	China	SD-01	*Saposhnikovia divaricata*	Neimenggu, China
PP-06	*P. praeruptorum*	China	SD-02	*S. divaricata*	Hebei, China
PP-07	*P. praeruptorum*	China	SD-03	*S. divaricata*	China
PP-08	*P. praeruptorum*	China	SD-04	*S. divaricata*	-
PP-09	*P. praeruptorum*	China	SD-05	*S. divaricata*	Neimenggu, China
PP-10	*P. praeruptorum*	China	SD-06	*S. divaricata*	Neimenggu, China
PP-11	*P. praeruptorum*	China	SD-07	*S. divaricata*	Neimenggu, China
PP-12	*P. praeruptorum*	China	SD-08	*S. divaricata*	Jilin, China
PP-13	*P. praeruptorum*	China	SD-09	*S. divaricata*	China
PP-14	*P. praeruptorum*	China	SD-10	*S. divaricata*	China

‘-’: unknown.

**Table 2:** List of standard reference samples used for the genetic identification in this study.

No.	Accession Code	Scientific Name	Medicinal Name
1	PR-PP01	*Peucedanum praeruptorum* Dunn	Peucedani Radix
2	PR-PP02		
3	PR-PP03		
4	PR-PP04		
5	PR-PP05		
6	PR-AD01	*Angelica decursiva* (Miq.) Franch. et Sav. (=*Peucedanum decursivum* Maxim.)	
7	PR-AD02		
8	PR-AD03		
9	PR-AD04		
10	PR-AD05		
11	PJR-PJ01	*Peucedanum japonicum* Thunberg	Peucedani Japonici Radix
12	PJR-PJ02		
13	PJR-PJ03		
14	PJR-PJ04		
15	PJR-PJ05		
16	SR-SD01	*Saposhnikovia divaricata* Schischkin	Saposhnikoviae Radix
17	SR-SD02		
18	SR-SD03		
19	SR-SD04		
20	SR-SD05		

**Table 3:** Amplicon size of plastid loci and nuclear barcode regions in species of Table 1 and Table 2 samples and sequence characteristics, namely single and different multi-locus combinations.

Barcode Target	Amplicon Size (~bp)	Aligned Length (bp)	Conserved Sites	Variable Sites	Parsimony Informative Sites	Singleton Site
ITS	700	689–693	648	44	2	42
*matk*	930	933	920	13	4	9
*rbcL*	670	670	664	6	1	5
*psbA-trnH*	320	311–345	323	17	none	16
*trnL-F* intergenic sapcer	440	444	440	4	none	4
*matk* + *rbcL*		1603	1584	19	5	14
*psbA-trnH* + *trnL-F* intergenic spacer		755–789	763	21	none	20
*mark*+*rbcL*+ *psbA-trnH*		1914–1948	1907	36	5	30
*Mark* + *rbcL*+ *trnL-F* intergenic spacer		2047	2024	23	5	18
Four plastid targets		2358–2392	2347	40	5	34

**Table 4:** Pearson’s correlation coefficients among PJ, PP, AD, and SD samples.

Sample	Value	PJ	PP	AD	SD
PJ	Mean	0.957			
	Median	0.966			
	Max	0.991			
	Min	0.849			
PP	Mean	0.029	0.791		
	Median	0.021	0.976		
	Max	0.321	0.999		
	Min	−0.058	−0.034		
AD	Mean	0.791	−0.011	0.872	
	Median	0.976	−0.014	0.932	
	Max	0.999	0.103	0.995	
	Min	−0.034	−0.057	0.563	
SD	Mean	−0.099	−0.038	−0.061	0.764
	Median	−0.097	−0.038	−0.060	0.822
	Max	−0.072	0.036	−0.044	0.982
	Min	−0.155	−0.079	−0.084	0.330

PJ: *Peucedanum japonicum*, PP: *P. praeruptorum*, AD: *Angelica decursiva*, SD: *Saposhnikovia divaricata*.

## Data Availability

Data sharing not applicable.

## Supplementary Materials

The following supporting information can be downloaded online, Figure S1: Chromatograms of the samples of *Peucedanum japonicum*, *P. praeruptorum*, *Angelica decursiva*, and *Saposhnikovia divaricata*; Figure S2: Multiple comparison of absolute peak areas among the samples; Figure S3: Silhouette plot; Figures S4–S7: Average Pearson’s correlation coefficients among the samples; Table S1: Sequence identity matrix; Table S2: Retention times and detection wavelengths of profiling peaks; Table S3: Precision of profiling peaks; Table S4: Pearson’s correlation coefficients among the samples.

## Sample Availability

Samples are not available from the authors.
