# Supplementary material for: Chemotaxonomic Classification of Peucedanum japonicum and Its Chemical Correlation with Peucedanum praeruptorum, Angelica decursiva, and Saposhnikovia divaricata by Liquid Chromatography Combined with Chemometrics
Source: Molecules. 2022 Mar 3;27(5):1675. doi: 10.3390/molecules27051675 (PMC8911569; doi:10.3390/molecules27051675)
Supplement: Supplementary file 1 [file molecules-27-01675-s001.zip › Supplement Materials.pdf]

Article

Chemotaxonomic Classification of *Peucedanum japonicum* and Its Chemical Correlation with *Peucedanum praeruptorum*, *Angelica decursiva*, and *Saposhnikovia divaricata* by Liquid Chromatography Combined with Chemometrics

Jung-Hoon Kim [1,†], Eui-Jeong Doh [2,†] and Guemsan Lee [2,3,*]

[1] Division of Pharmacology, School of Korean Medicine, Pusan National University, Yangsan 50612, Korea; kmsct@pusan.ac.kr
[2] Research Center of Traditional Korean Medicine, Wonkwang University, Iksan 54538, Korea; bluemoon-lion@hanmail.net
[3] Department of Herbology, College of Korean Medicine, Wonkwang University, Iksan 54538, Korea
* Correspondence: rasfin@wku.ac.kr; Tel.: +82-63-850-6985
† These authors contributed equally to this work.

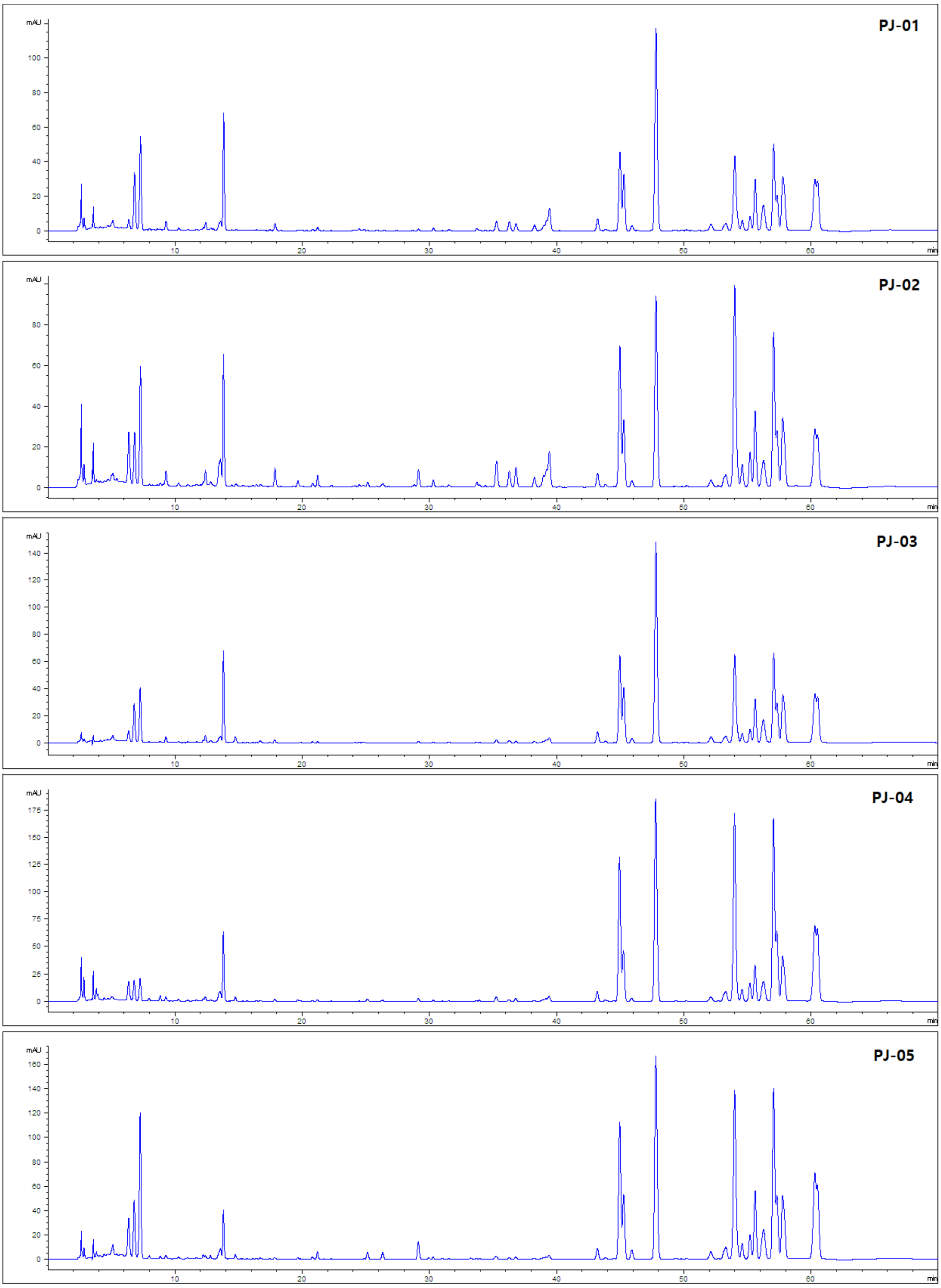

Figure S1. Chromatograms of the samples of *Peucedanum japonicum* (PJ01–16), *P. praeruptorum* (PP01–27), *Angelica decursiva* (AD01–07), and *Saposhnikovia divaricata* (SD01–10) at a detection wavelength of 325 nm.

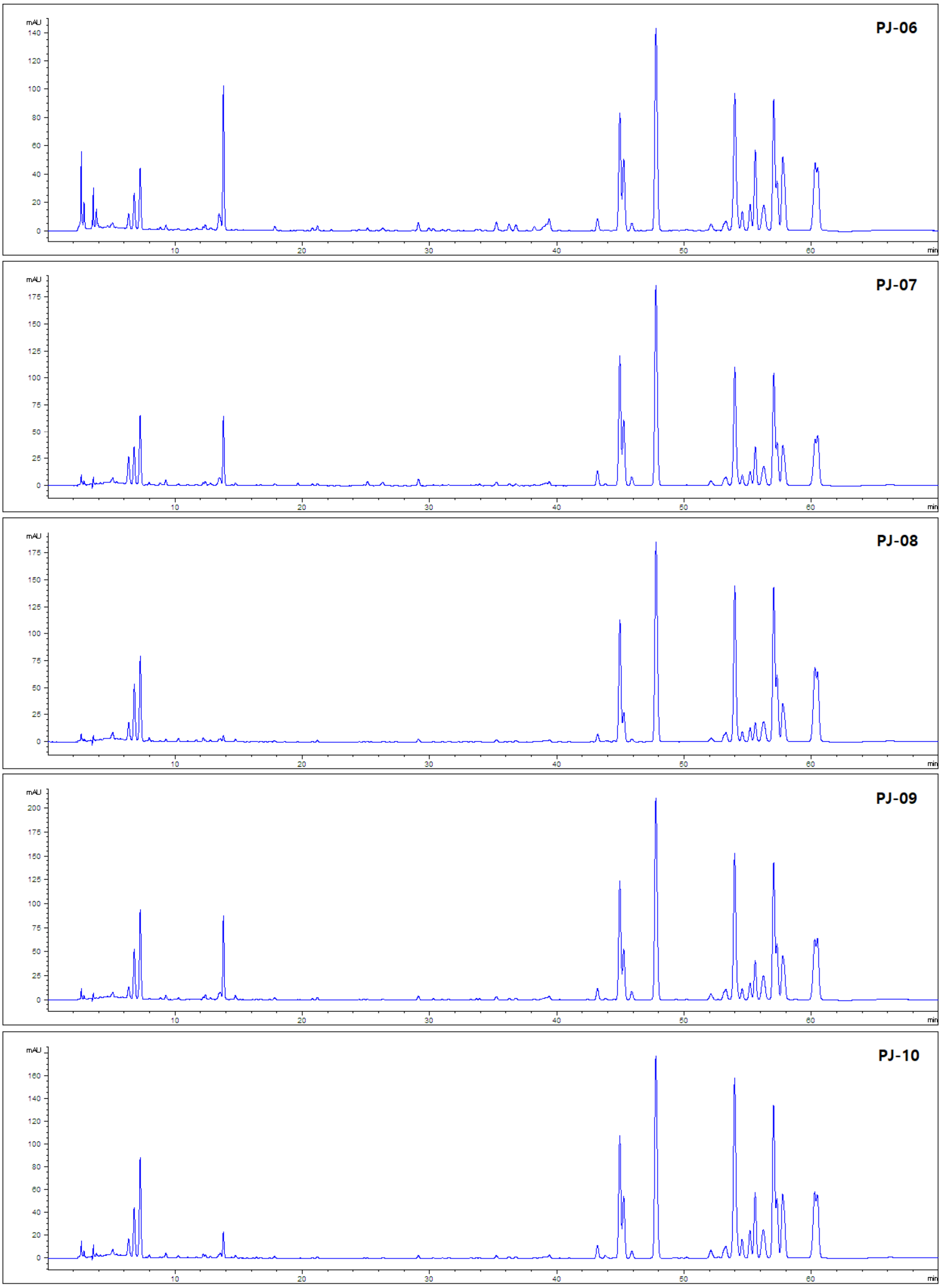

Continued.

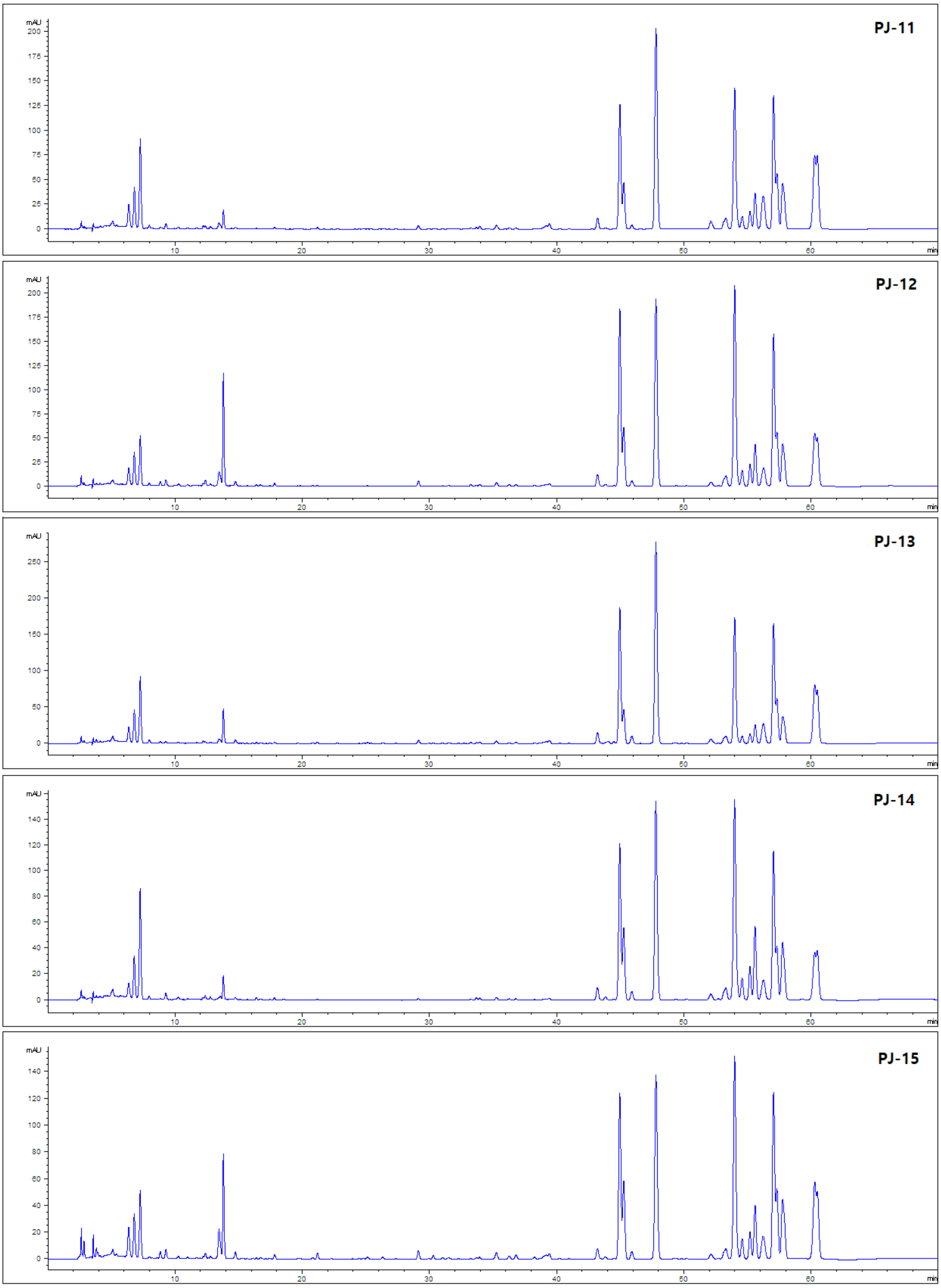

Continued.

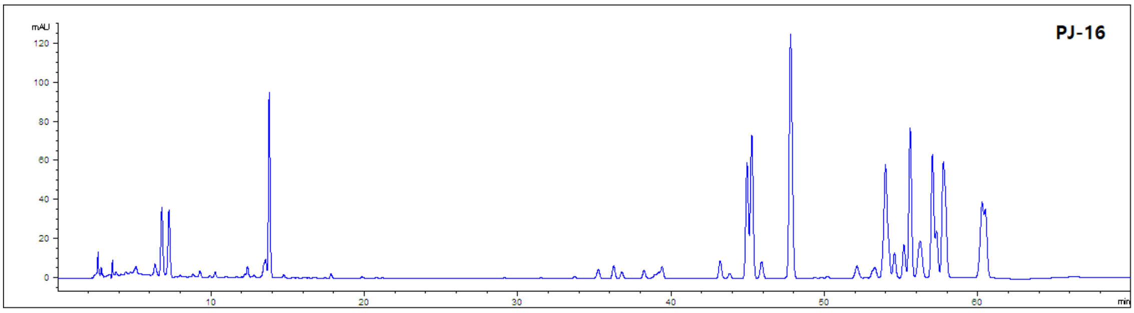

Continued.

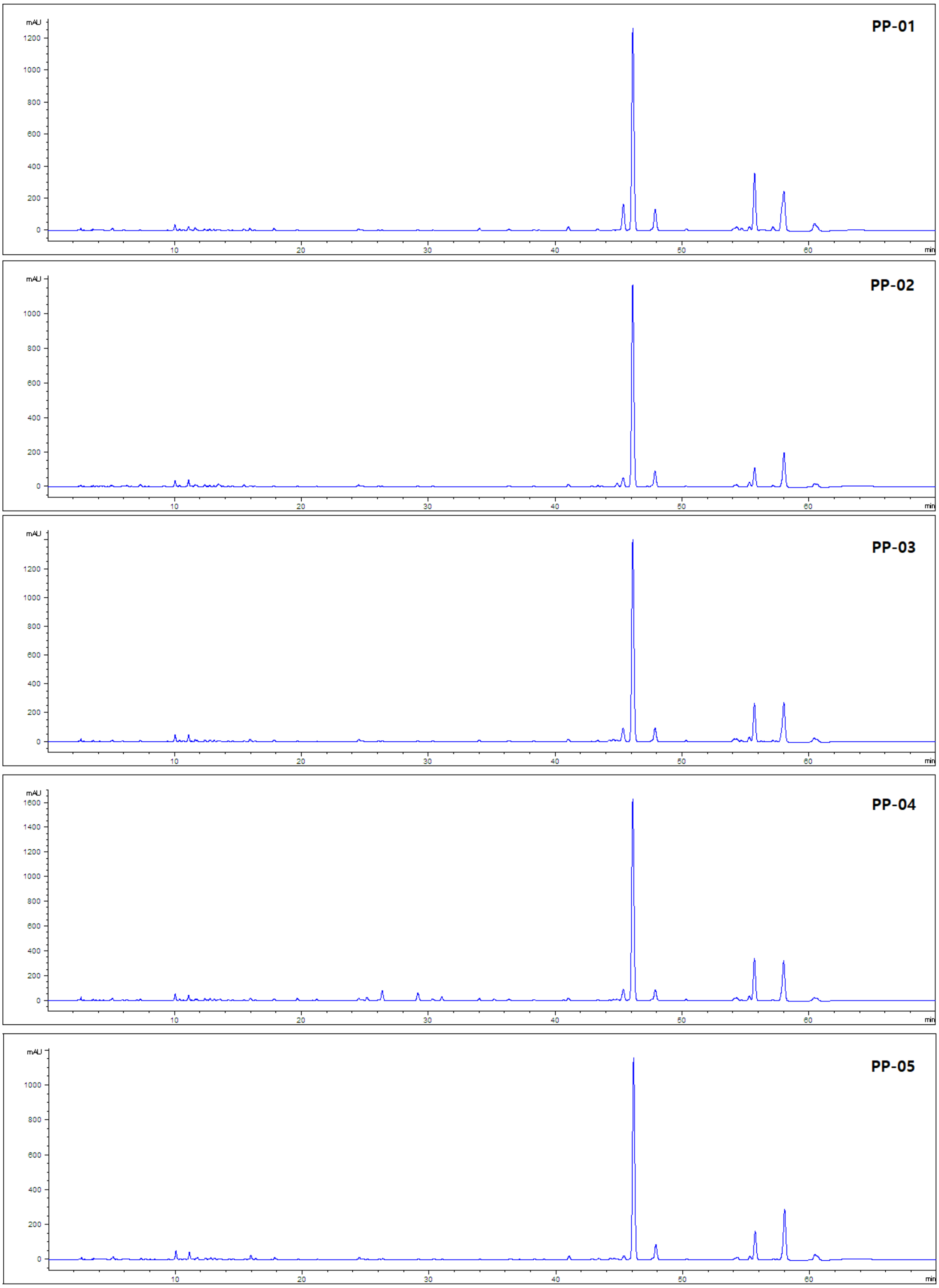

Continued.

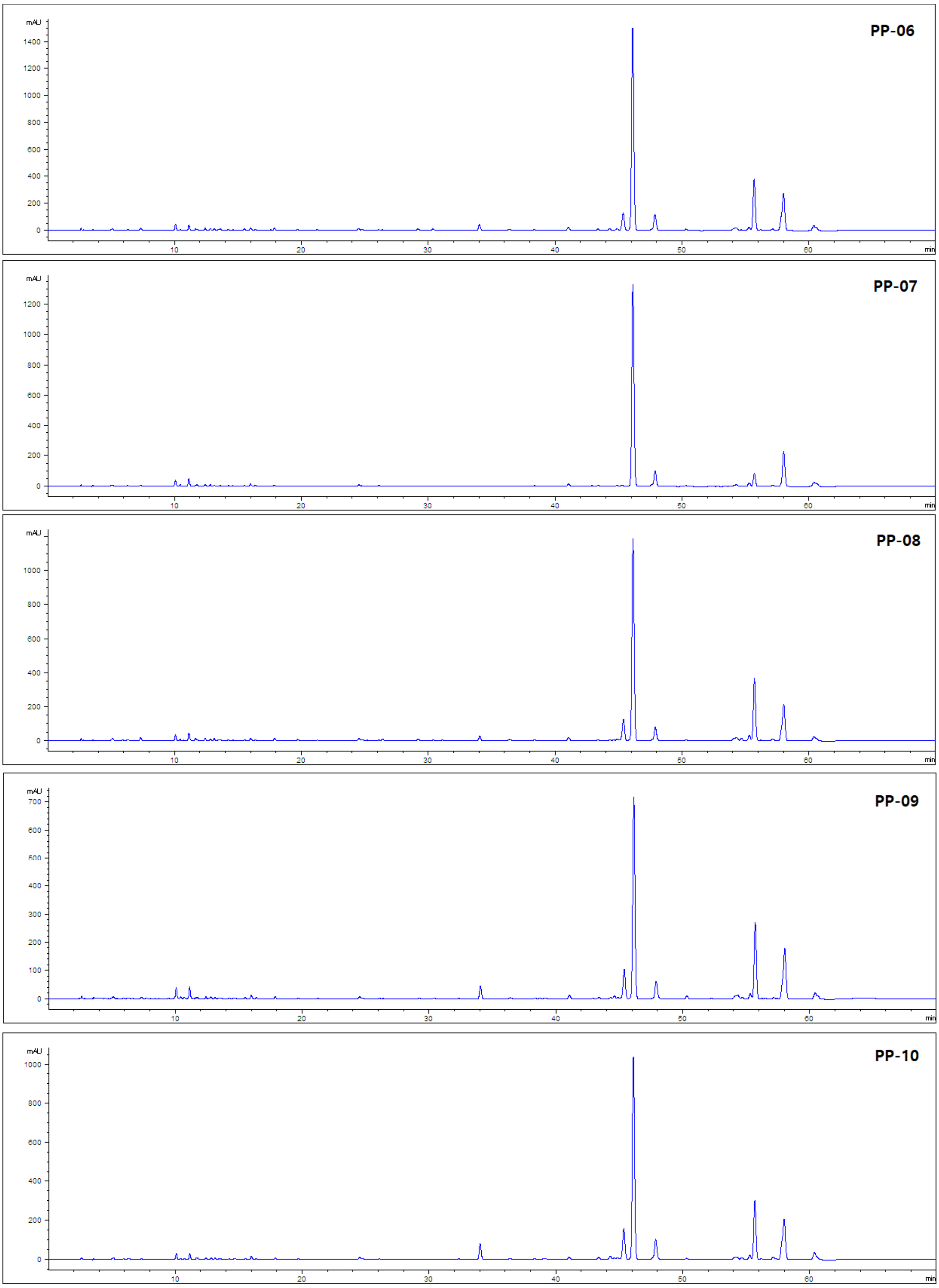

Continued.

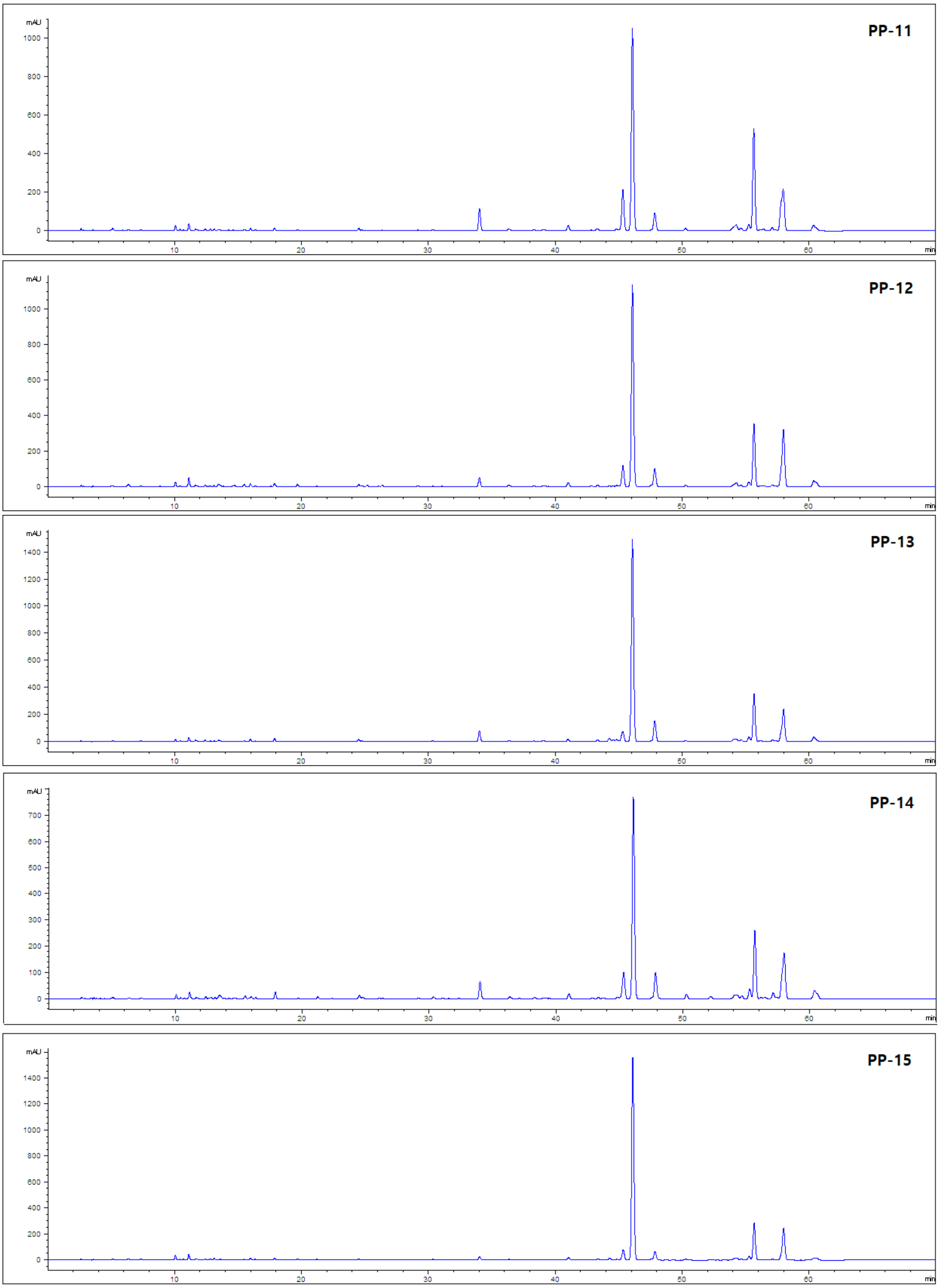

Continued.

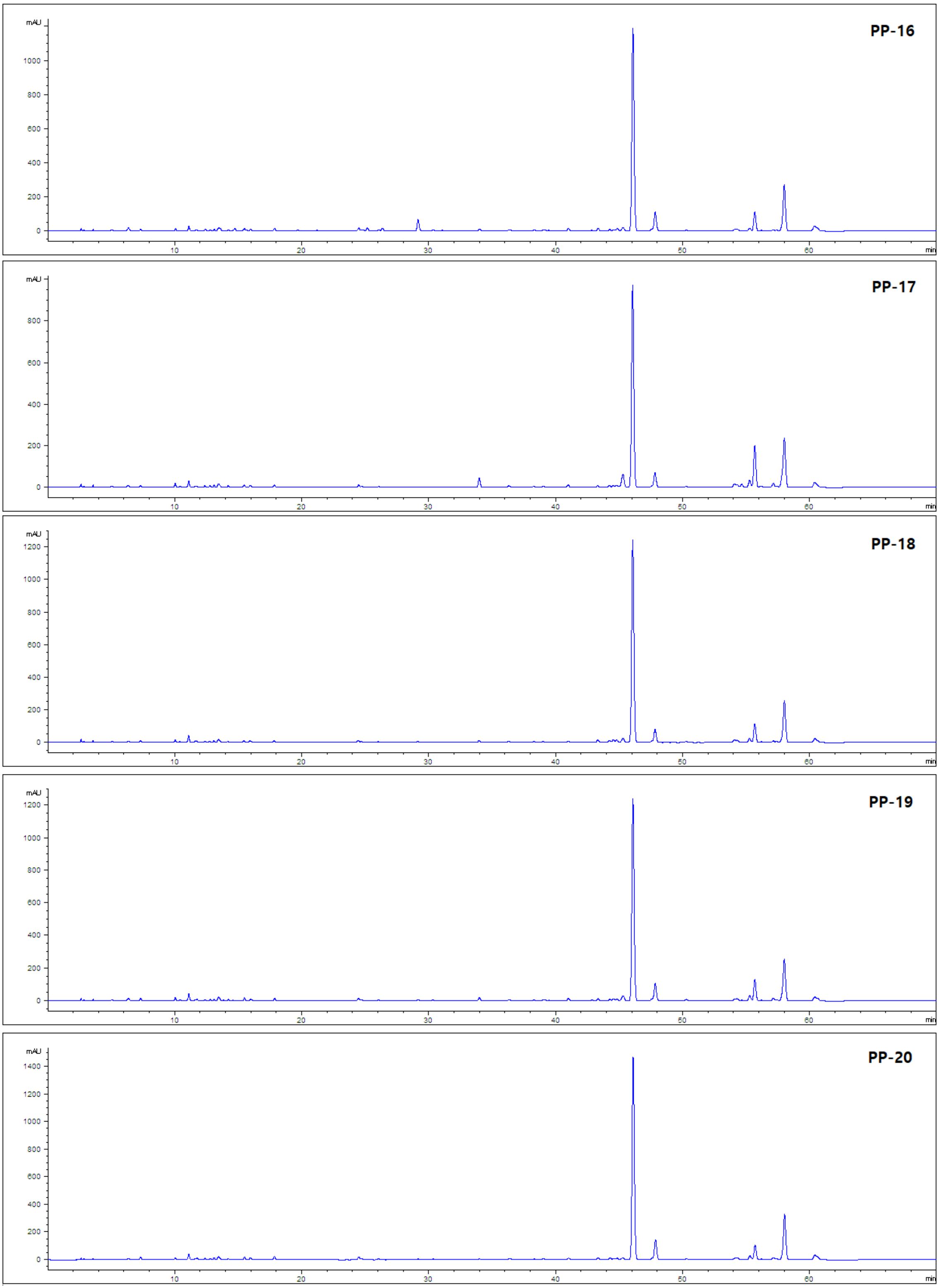

Continued.

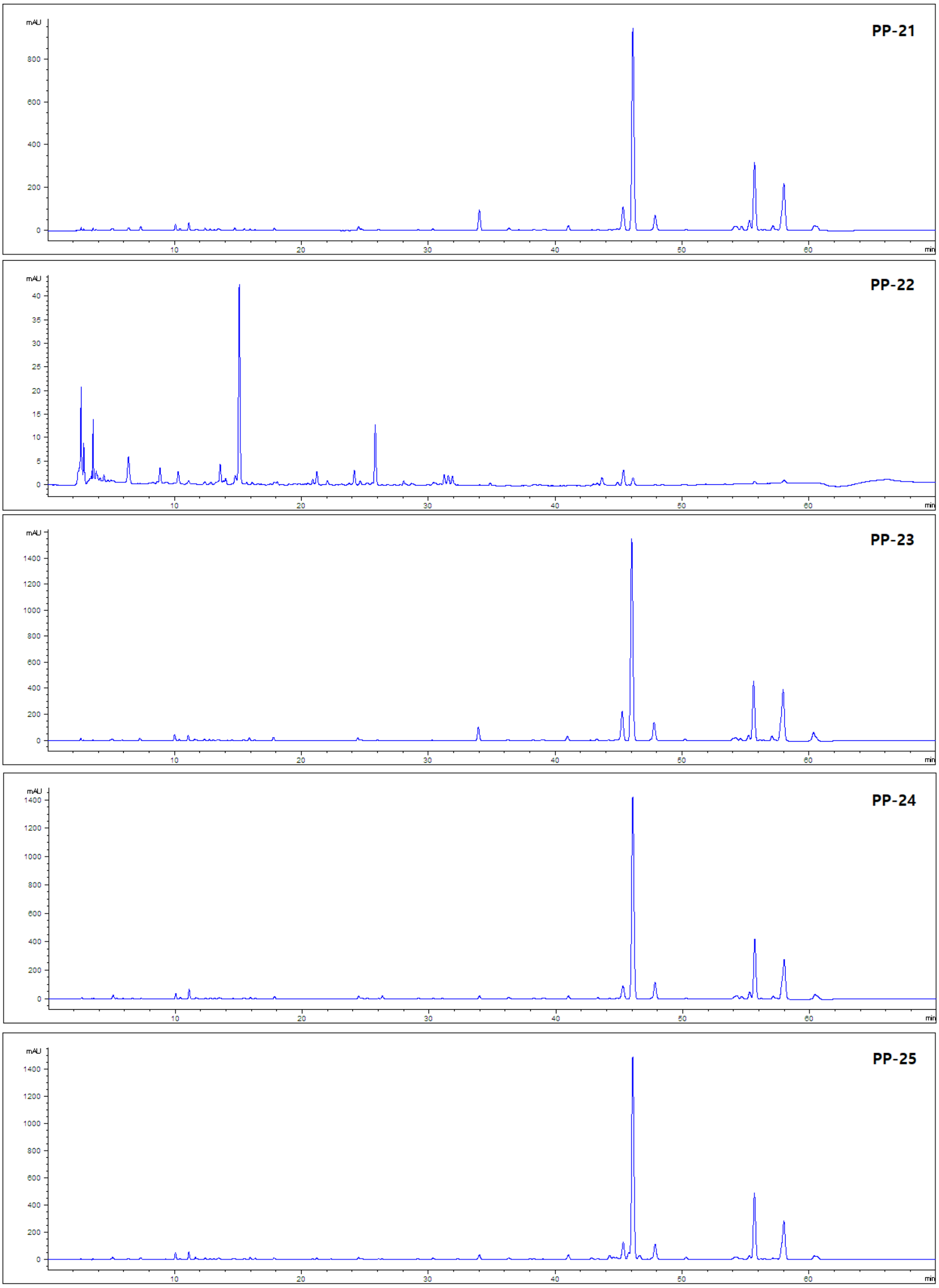

Continued.

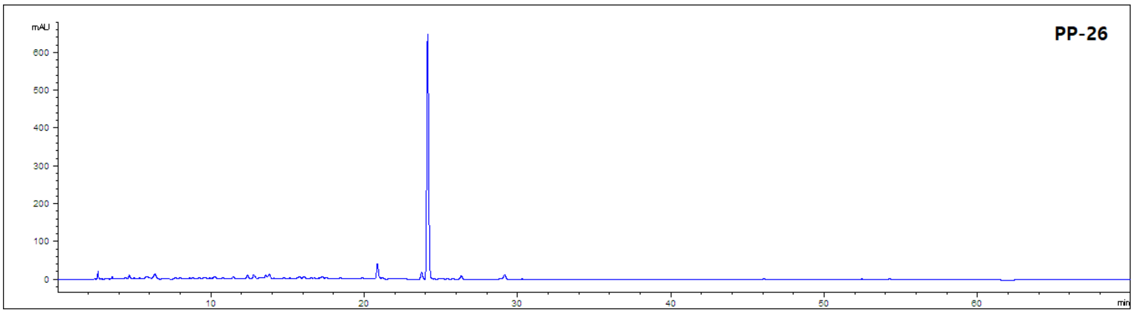
PP-26
mAU
600
500
400
300
200
100
0
10
20
30
40
50
60
min
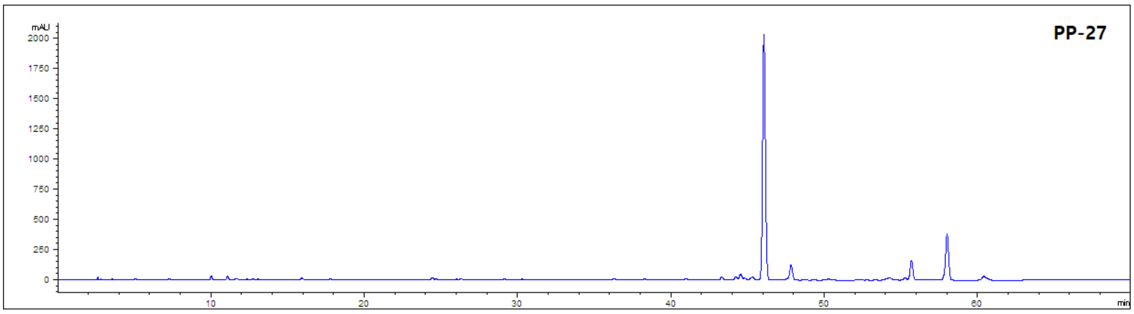
PP-27
mAU
2000
1750
1500
1250
1000
750
500
250
0
10
20
30
40
50
60
min

Continued.

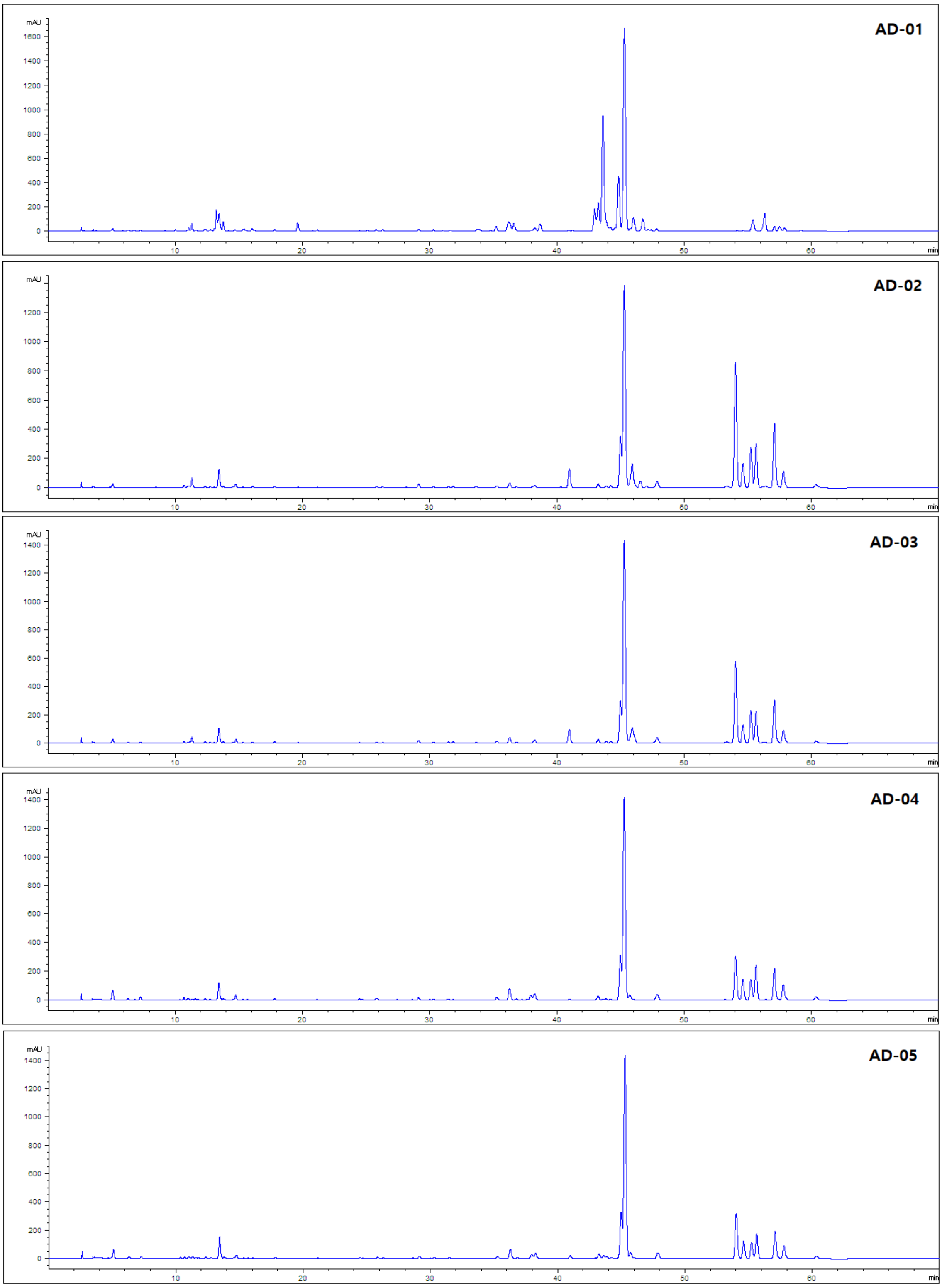

Continued.

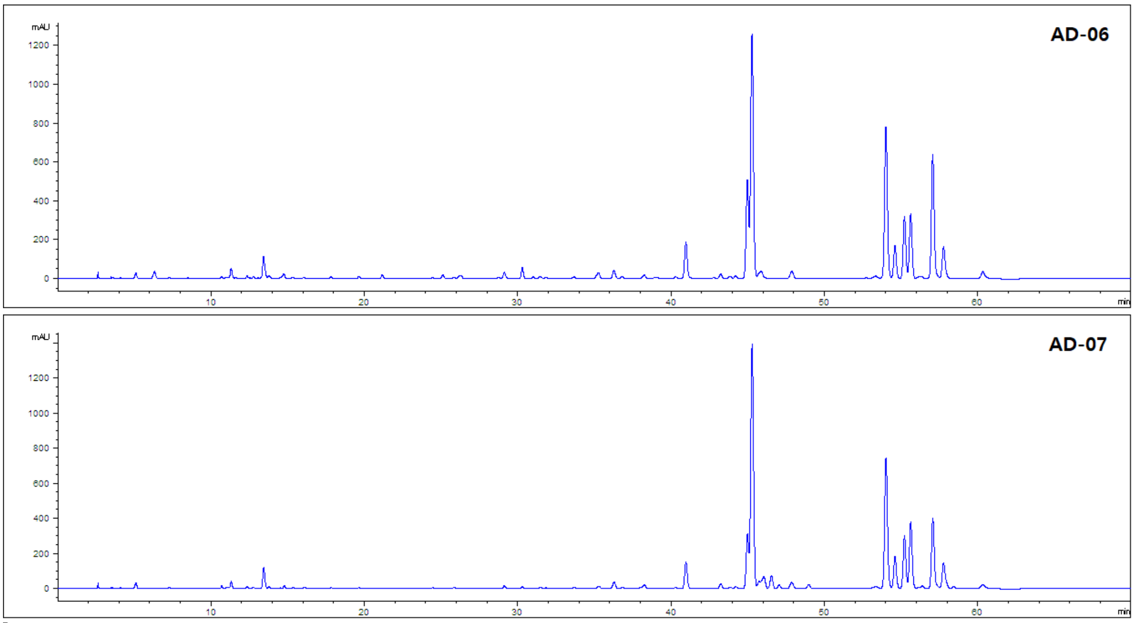

Continued.

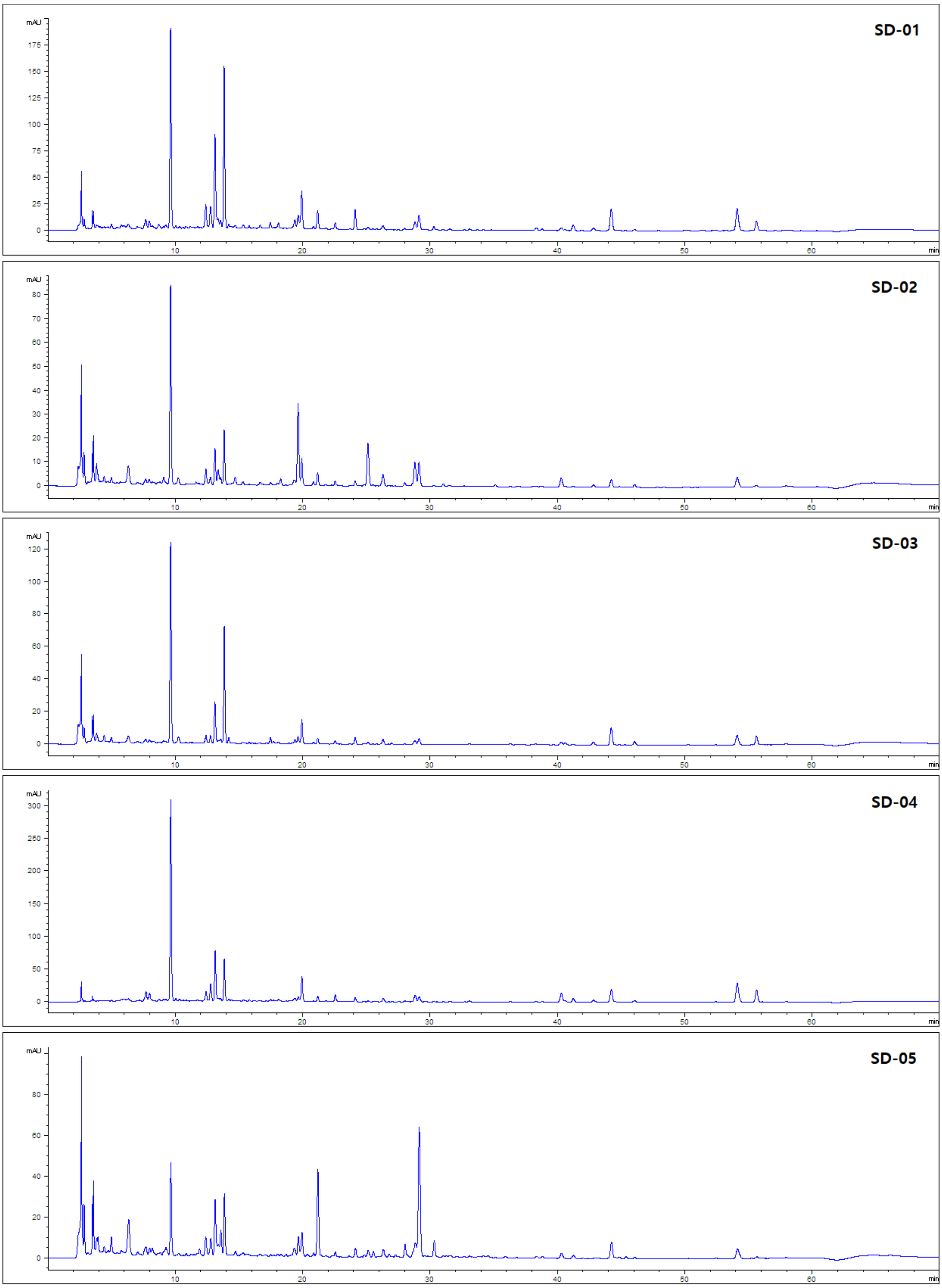

Continued.

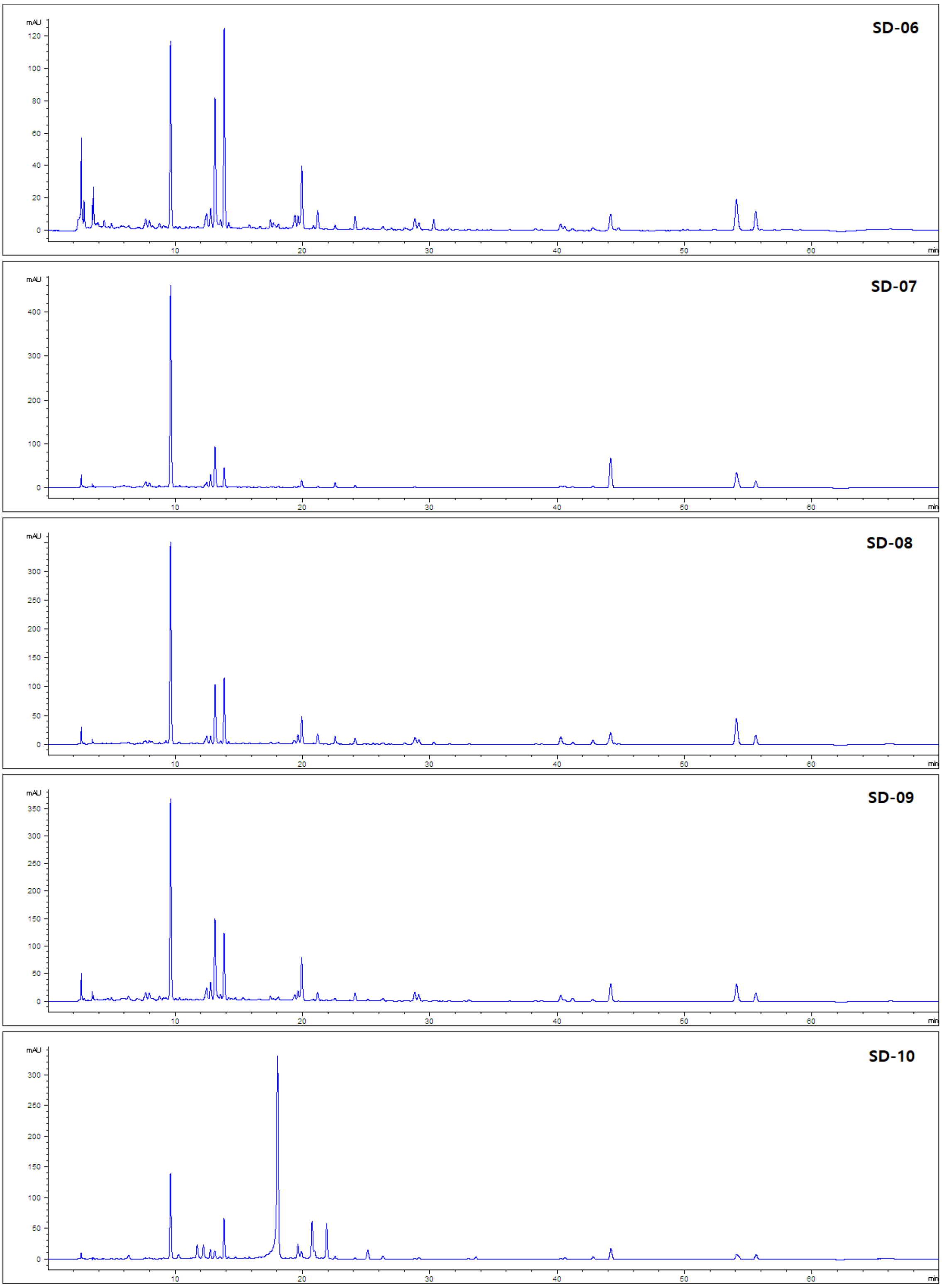

Continued.

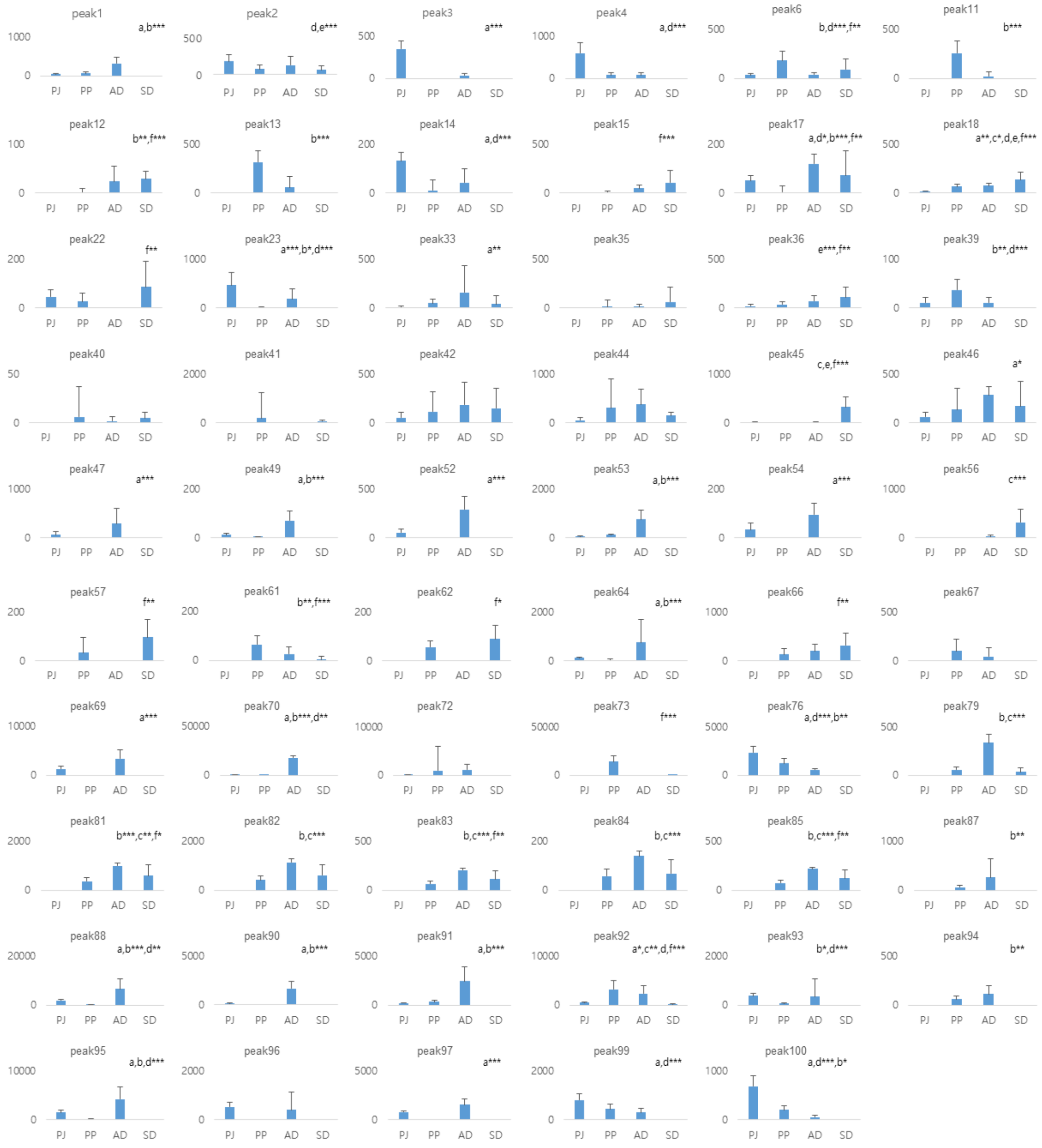

Figure S2. Multiple comparison of absolute areas of profiling peaks from *P. japonicum* (PJ), *P. praeruptorum* (PP), *A. decursiva* (AD), and *S. divaricata* (SD) samples. Difference in peaks areas among the samples was compared using the Tukey's test, with significance at $^{*}p < 0.05$, $^{**}p < 0.01$, and $^{***}p < 0.001$. (a) PJ and AD, (b) PP and AD, (c) AD and SD, (d) PP and PJ, (e) PJ and SD, and (f) PP and SD.

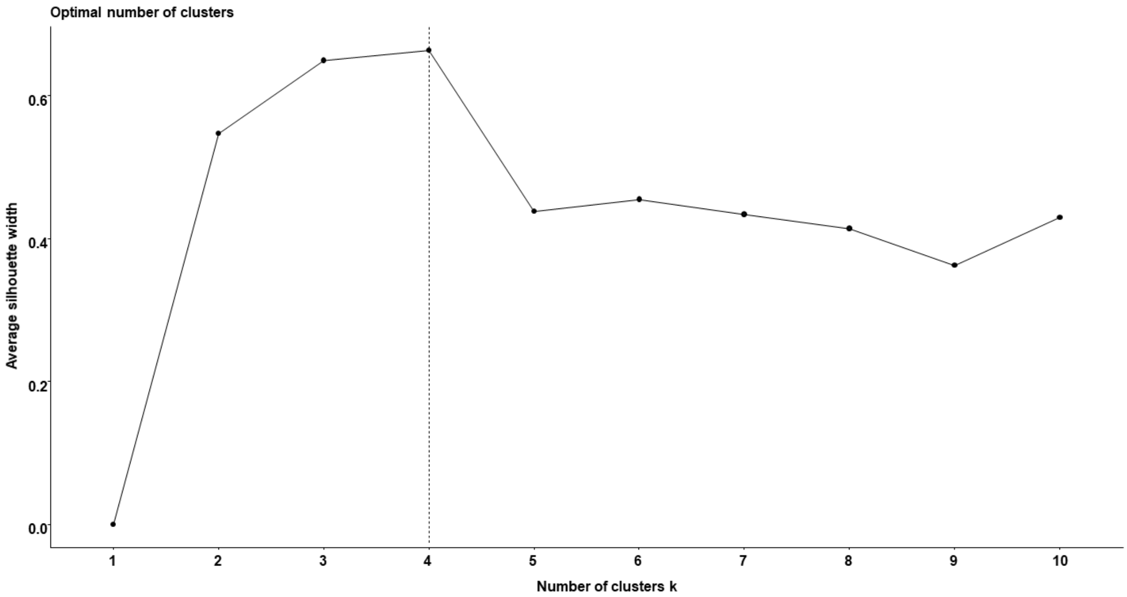

Figure S3. Silhouette plot for the selection of optimal number of clusters in *k*-means clustering.

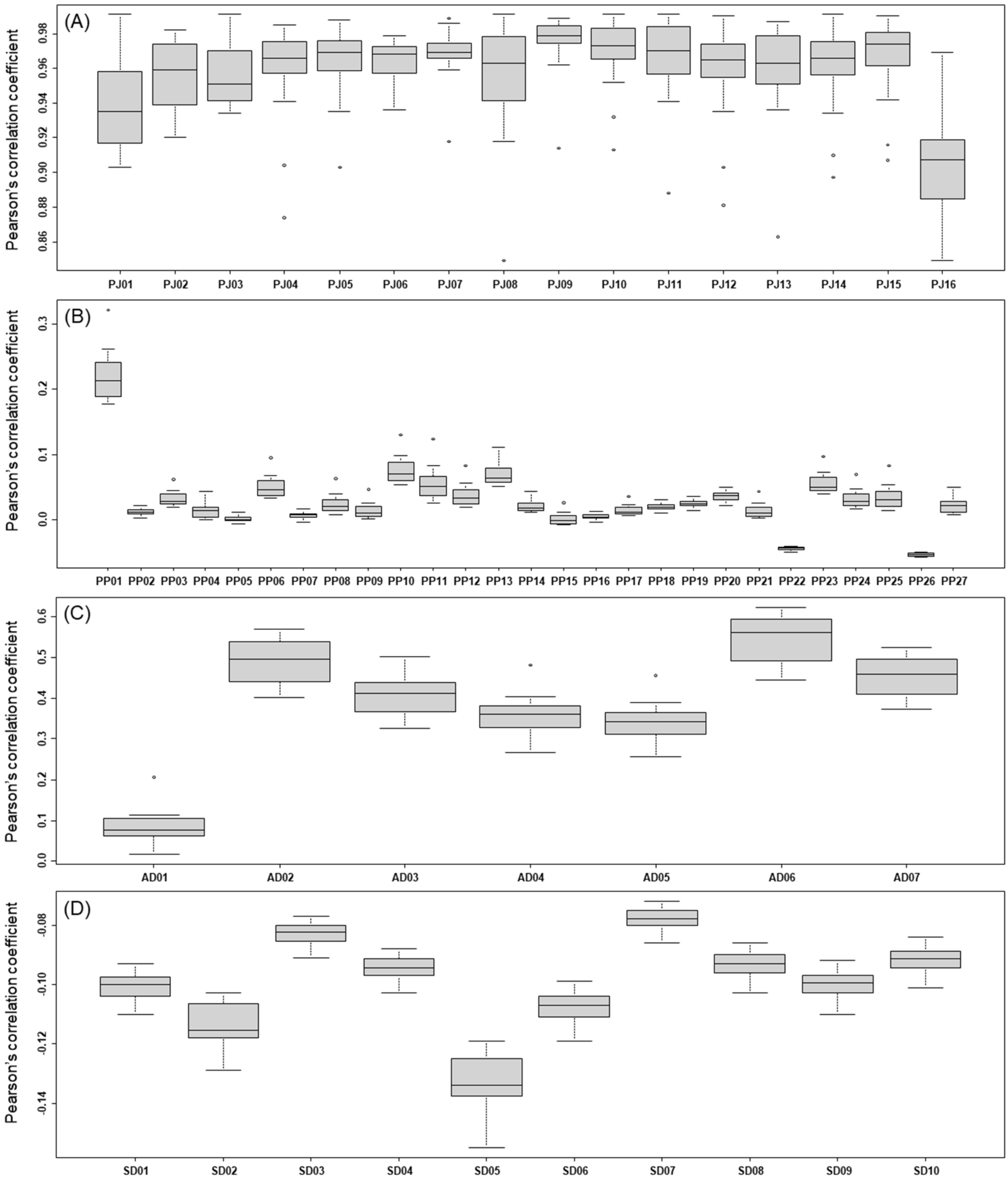

Figure S4. Average Pearson's correlation coefficients of *Peucedanum japonicum* samples (PJ) to other species samples with PJ (A), PP (B), AD (C), and SD (D).

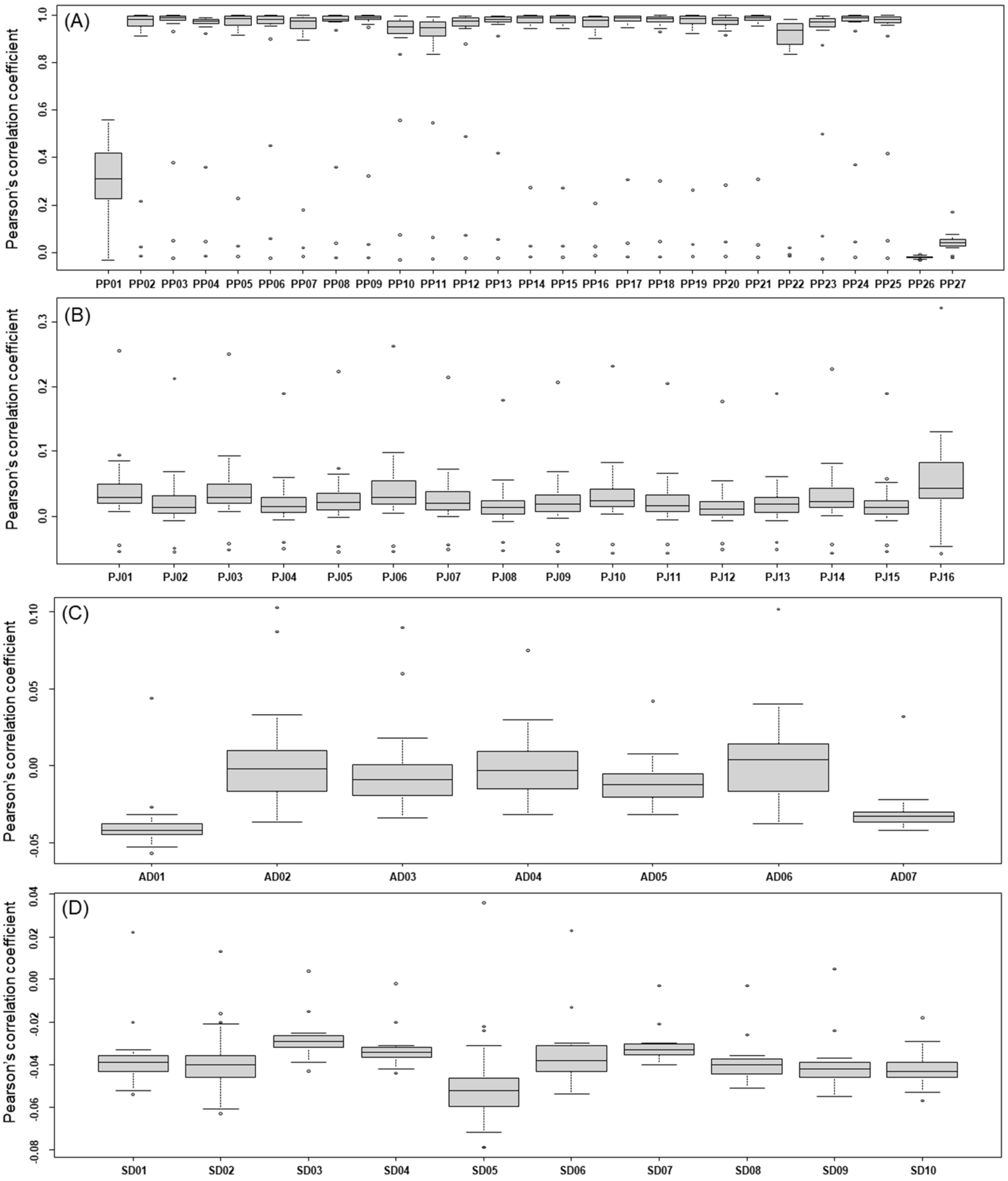

Figure S5. Average Pearson's correlation coefficients of *P. praeruptorum* samples (PP) to other species samples with PP (A), PJ (B), AD (C), and SD (D).

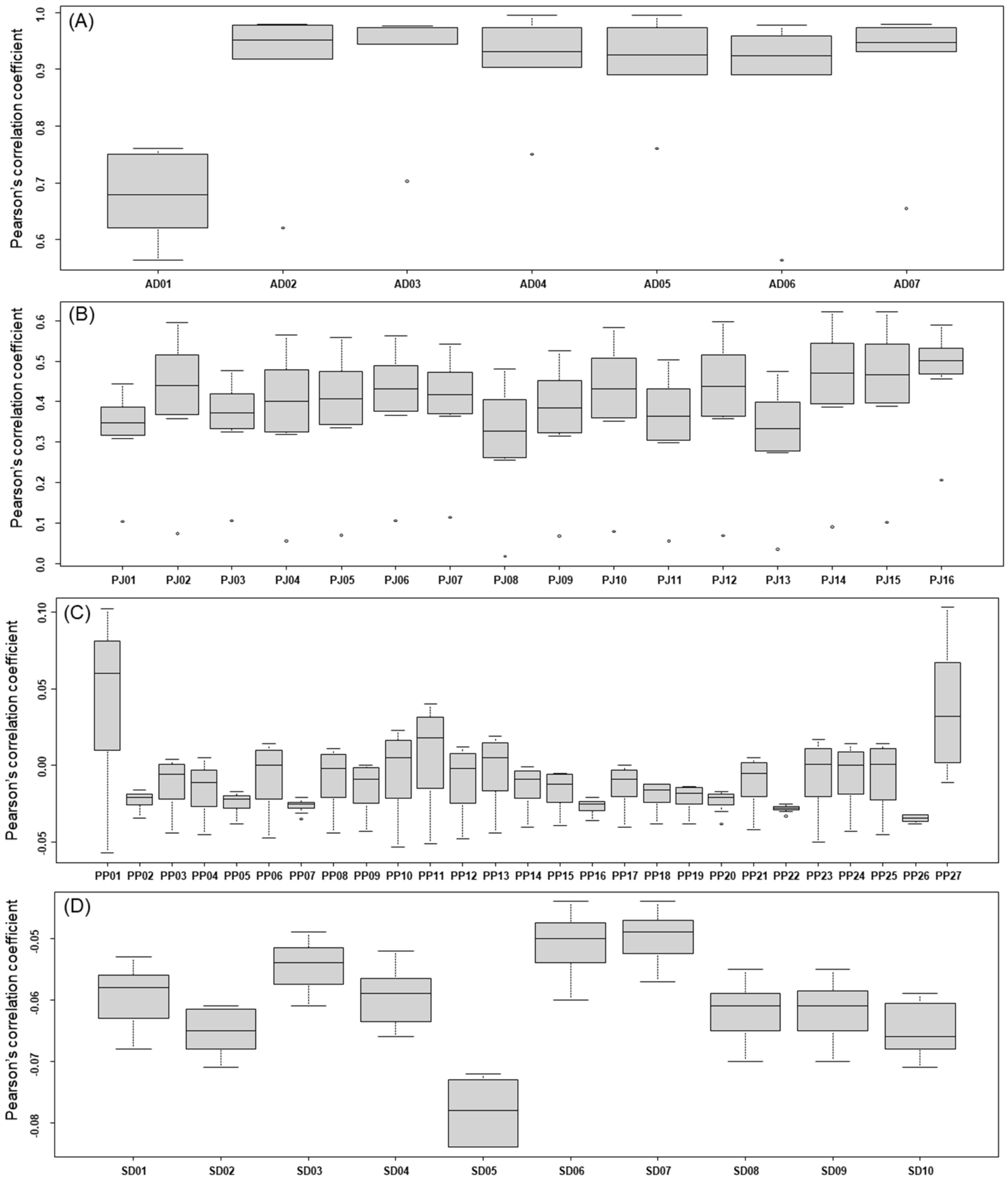

Figure S6. Average Pearson's correlation coefficients of *A. decursiva* samples (AD) to other species samples with AD (A), PJ (B), PP (C), and SD (D).

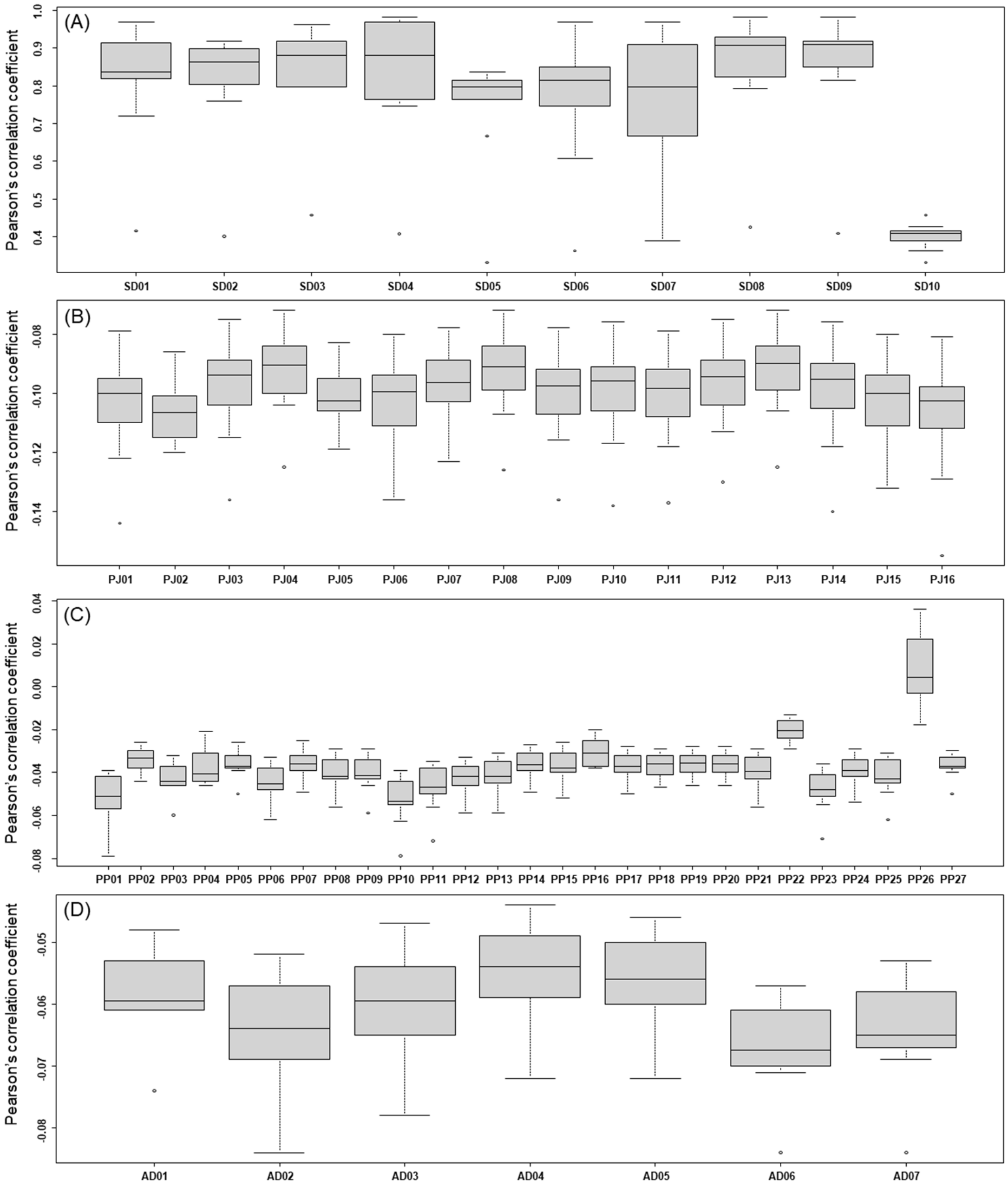

Figure S7. Average Pearson's correlation coefficients of *S. divaricata* samples (SD) to other species samples with SD (A), PJ (B), PP (C), and AD (D).

Table S1. The sequence identity matrix (excel file attached)

Table S2. Retention times and detection wavelengths of profiling peaks in the samples

Peak No.	Retention time (min)	Detection wavelength (nm)	Peak No.	Retention time (min)	Detection wavelength (nm)
peak1	5.07	310	peak51	34.59	250
peak2	6.34	325	peak52	35.25	325
peak3	6.79	325	peak53	36.28	325
peak4	7.25	325	peak54	36.78	325
peak5	7.95	275	peak55	37.92	310
peak6	8.58	275	peak56	40.28	250
peak7	8.80	325	peak57	40.58	250
peak8	9.21	325	peak58	40.97	350
peak9	9.23	310	peak59	41.02	310
peak10	9.60	300	peak60	41.21	250
peak11	10.01	335	peak61	42.81	310
peak12	10.31	325	peak62	42.83	275
peak13	11.07	335	peak63	42.94	325
peak14	11.35	275	peak64	43.20	325
peak15	11.78	275	peak65	43.59	325
peak16	11.81	235	peak66	44.20	335
peak17	12.37	310	peak67	44.53	325
peak18	12.74	325	peak68	44.82	325
peak19	13.10	300	peak69	44.95	325
peak20	13.22	335	peak70	45.26	325
peak21	13.37	325	peak71	45.35	325
peak22	13.58	325	peak72	45.86	325
peak23	13.80	335	peak73	46.05	325
peak24	13.81	300	peak74	46.52	325
peak25	14.67	325	peak75	47.02	325
peak26	14.76	350	peak76	47.86	325
peak27	15.08	325	peak77	48.37	275
peak28	15.65	325	peak78	48.64	250
peak29	15.91	335	peak79	48.67	235
peak30	17.70	275	peak80	48.96	325
peak31	18.02	275	peak81	49.00	235
peak32	19.37	300	peak82	49.31	235
peak33	19.60	335	peak83	51.20	275
peak34	19.90	250	peak84	51.77	275
peak35	20.84	325	peak85	52.27	275
peak36	21.15	325	peak86	53.25	250
peak37	21.87	275	peak87	53.31	275
peak38	22.53	250	peak88	54.05	325
peak39	22.65	275	peak89	54.09	250
peak40	23.69	310	peak90	54.57	325
peak41	24.10	325	peak91	55.25	325
peak42	25.11	250	peak92	55.59	325
peak43	25.79	310	peak93	56.23	325
peak44	26.29	250	peak94	56.39	325
peak45	28.80	250	peak95	57.04	325
peak46	29.12	310	peak96	57.31	325
peak47	32.00	250	peak97	57.76	325
peak48	33.22	310	peak98	58.00	325
peak49	33.69	325	peak99	60.23	325
peak50	34.01	325	peak100	60.49	325

Peak 10: prim-O-glucosyl-cimifugin, peak 19: Cimifugin, peak 21: umbelliferone, peak 34: sec-O-glucosyl-hamaudol, peak 42: psoralen, peak 44: xanthotoxin, peak 46: bergapten, peak 48: oxypeucedanin, peak 57: imperatorinpeak, peak 66: decursin, peak 73: praeruptorin A, 92: praeruptorin B, peak 98: praeruptorin C.

Table S3. Intra- and interday precisions of profiling peaks in *Peucedanum japonicum* sample (PJ11)

Peak No.	Intraday precision (n = 3)				Interday precision (n = 3 × 3)			
	Retention time (min)		Absolute peak area		Retention time (min)		Absolute peak area	
	AVR	RSD (%)	AVR	RSD (%)	AVR	RSD (%)	AVR	RSD (%)
Peak 2	6.33	0.09	61.73	1.51	6.33	0.05	60.87	0.96
Peak 3	6.81	0.18	203.53	2.25	6.80	0.09	200.70	0.23
Peak 4	7.27	0.12	380.73	0.77	7.26	0.06	379.00	0.20
Peak 6	8.52	0.08	83.97	1.59	8.52	0.04	83.27	0.25
Peak 9	9.25	0.07	19.63	0.78	9.25	0.05	19.53	0.30
Peak 14	11.31	0.06	8.37	1.38	11.31	0.06	8.17	1.87
Peak 16	11.82	0.04	72.70	0.41	11.82	0.01	72.53	0.21
Peak 17	12.38	0.04	19.93	2.47	12.37	0.01	19.87	1.05
Peak 23	13.80	0.05	115.60	0.69	13.79	0.01	115.03	0.22
Peak 36	21.18	0.02	13.13	0.44	21.18	0.02	13.00	0.77
Peak 42	25.14	0.01	8.85	0.52	25.13	0.02	8.73	2.32
Peak 44	26.33	0.01	16.67	1.25	26.32	0.02	16.73	0.91
Peak 46	29.14	0.01	17.70	0.98	29.13	0.02	17.53	0.66
Peak 47	32.05	0.01	105.43	0.90	32.05	0.01	104.97	0.22
Peak 48	33.26	0.02	10.03	3.89	33.26	0.01	8.80	2.53
Peak 51	34.65	0.02	42.87	0.88	34.65	0.01	42.80	0.23
Peak 64	43.27	0.03	57.47	2.02	43.28	0.03	56.90	0.35
Peak 69	45.03	0.03	562.17	1.03	45.04	0.03	558.77	0.10
Peak 71	45.33	0.03	306.50	1.16	45.34	0.03	304.27	0.05
Peak 72	45.95	0.02	54.70	1.93	45.96	0.03	53.97	0.21
Peak 76	47.87	0.02	1021.80	0.77	47.89	0.03	1017.20	0.07
Peak 78	48.72	0.02	79.30	0.55	48.74	0.04	79.40	0.70
Peak 88	54.09	0.02	778.43	0.75	54.10	0.04	776.77	0.29
Peak 90	54.67	0.02	84.90	0.74	54.68	0.04	85.53	1.17
Peak 91	55.29	0.02	120.20	0.87	55.30	0.04	120.67	0.70
Peak 92	55.70	0.02	383.30	0.86	55.70	0.04	382.97	0.34
Peak 93	56.34	0.02	194.53	1.18	56.35	0.04	196.10	0.84
Peak 95	57.15	0.02	645.67	0.85	57.16	0.04	644.03	0.29
Peak 96	57.42	0.02	226.57	0.74	57.42	0.04	225.13	0.18
Peak 97	57.88	0.02	467.97	0.88	57.88	0.04	464.80	0.28
Peak 99	60.42	0.03	340.33	0.74	60.43	0.04	338.13	0.12
Peak 100	60.64	0.03	326.90	0.88	60.64	0.04	325.47	0.26

Peak 42: psoralen, peak 44: xanthotoxin, peak 46: bergapten, peak 48: oxypeucedanin, peak 92: praeruptorin B

Table S4. Pearson's correlation coefficients among the samples

	PJ01	PJ02	PJ03	PJ04	PJ05	PJ06	PJ07	PJ08	PJ09	PJ10	PJ11	PJ12	PJ13	PJ14	PJ15	PJ16	PP01	PP02	PP03	PP04
PJ01	1.000																			
PJ02	0.930	1.000																		
PJ03	0.991	0.939	1.000																	
PJ04	0.904	0.956	0.941	1.000																
PJ05	0.935	0.974	0.946	0.962	1.000															
PJ06	0.969	0.974	0.979	0.958	0.963	1.000														
PJ07	0.963	0.959	0.982	0.966	0.969	0.971	1.000													
PJ08	0.918	0.939	0.944	0.978	0.975	0.936	0.967	1.000												
PJ09	0.962	0.973	0.977	0.979	0.983	0.978	0.989	0.984	1.000											
PJ10	0.932	0.975	0.952	0.973	0.988	0.968	0.970	0.978	0.986	1.000										
PJ11	0.941	0.951	0.961	0.973	0.983	0.956	0.978	0.991	0.988	0.985	1.000									
PJ12	0.903	0.973	0.935	0.981	0.955	0.960	0.969	0.954	0.975	0.966	0.957	1.000								
PJ13	0.936	0.937	0.963	0.973	0.963	0.944	0.986	0.986	0.985	0.965	0.987	0.965	1.000							
PJ14	0.910	0.975	0.934	0.962	0.976	0.954	0.966	0.961	0.974	0.991	0.970	0.976	0.958	1.000						
PJ15	0.916	0.982	0.942	0.985	0.976	0.974	0.968	0.963	0.981	0.981	0.968	0.990	0.960	0.978	1.000					
PJ16	0.954	0.920	0.951	0.874	0.903	0.969	0.918	0.849	0.914	0.913	0.888	0.881	0.863	0.897	0.907	1.000				
PP01	0.255	0.212	0.250	0.189	0.223	0.262	0.214	0.179	0.206	0.231	0.204	0.177	0.189	0.227	0.189	0.321	1.000			
PP02	0.020	0.002	0.022	0.010	0.008	0.015	0.014	0.012	0.012	0.013	0.012	0.004	0.015	0.011	0.002	0.020	0.217	1.000		
PP03	0.041	0.026	0.042	0.024	0.030	0.044	0.030	0.021	0.027	0.037	0.026	0.020	0.024	0.036	0.021	0.062	0.378	0.984	1.000	
PP04	0.020	0.013	0.020	0.006	0.021	0.026	0.016	0.000	0.007	0.017	0.004	0.001	0.003	0.017	0.004	0.043	0.359	0.971	0.986	1.000
PP05	0.007	-0.007	0.009	-0.001	-0.002	0.005	0.001	-0.001	-0.001	0.003	-0.001	-0.006	0.001	0.001	-0.006	0.012	0.228	0.998	0.987	0.975
PP06	0.065	0.045	0.064	0.037	0.050	0.068	0.048	0.033	0.043	0.056	0.042	0.033	0.038	0.056	0.036	0.095	0.450	0.965	0.995	0.981
PP07	0.014	-0.004	0.016	0.006	0.002	0.008	0.009	0.009	0.007	0.007	0.008	0.001	0.012	0.005	-0.001	0.010	0.180	0.998	0.977	0.963
PP08	0.033	0.022	0.032	0.013	0.025	0.039	0.020	0.008	0.017	0.030	0.015	0.011	0.011	0.031	0.014	0.063	0.358	0.981	0.996	0.986
PP09	0.022	0.010	0.022	0.005	0.012	0.026	0.010	0.001	0.008	0.019	0.007	0.003	0.004	0.019	0.005	0.047	0.322	0.988	0.996	0.982
PP10	0.094	0.068	0.093	0.060	0.074	0.098	0.072	0.055	0.068	0.082	0.066	0.054	0.061	0.081	0.058	0.130	0.557	0.922	0.972	0.953
PP11	0.068	0.056	0.065	0.036	0.056	0.082	0.046	0.025	0.043	0.065	0.039	0.035	0.029	0.068	0.041	0.124	0.545	0.912	0.964	0.950
PP12	0.051	0.032	0.050	0.025	0.037	0.056	0.035	0.020	0.030	0.043	0.028	0.021	0.024	0.043	0.024	0.083	0.489	0.953	0.990	0.977
PP13	0.085	0.061	0.085	0.056	0.065	0.086	0.068	0.053	0.063	0.074	0.061	0.051	0.059	0.073	0.052	0.111	0.418	0.971	0.994	0.979
PP14	0.028	0.013	0.029	0.015	0.018	0.029	0.019	0.013	0.017	0.024	0.016	0.011	0.016	0.023	0.011	0.043	0.273	0.995	0.991	0.978
PP15	0.007	-0.002	0.007	-0.005	0.000	0.011	-0.001	-0.008	-0.003	0.005	-0.005	-0.007	-0.006	0.006	-0.006	0.026	0.271	0.994	0.993	0.981
PP16	0.011	-0.004	0.013	0.004	0.003	0.007	0.008	0.005	0.004	0.004	0.004	-0.003	0.009	0.002	-0.004	0.009	0.207	0.997	0.980	0.974
PP17	0.020	0.009	0.022	0.011	0.013	0.023	0.012	0.008	0.012	0.019	0.011	0.007	0.010	0.019	0.007	0.036	0.307	0.994	0.996	0.981
PP18	0.026	0.010	0.029	0.016	0.016	0.024	0.020	0.018	0.018	0.022	0.018	0.011	0.020	0.020	0.010	0.030	0.302	0.993	0.992	0.976
PP19	0.034	0.014	0.036	0.022	0.021	0.029	0.027	0.024	0.024	0.026	0.024	0.016	0.028	0.024	0.014	0.034	0.263	0.998	0.990	0.975
PP20	0.047	0.022	0.050	0.033	0.030	0.038	0.040	0.038	0.037	0.036	0.037	0.025	0.042	0.032	0.023	0.041	0.284	0.992	0.987	0.971
PP21	0.020	0.010	0.020	0.006	0.013	0.025	0.010	0.002	0.008	0.019	0.007	0.004	0.004	0.020	0.006	0.043	0.309	0.989	0.994	0.980
PP22	-0.045	-0.049	-0.042	-0.040	-0.047	-0.046	-0.044	-0.040	-0.043	-0.043	-0.043	-0.042	-0.040	-0.043	-0.045	-0.047	0.021	0.977	0.930	0.921
PP23	0.068	0.049	0.068	0.045	0.056	0.073	0.051	0.041	0.049	0.062	0.048	0.039	0.044	0.061	0.043	0.097	0.498	0.950	0.988	0.970
PP24	0.041	0.028	0.041	0.022	0.032	0.047	0.029	0.017	0.025	0.037	0.023	0.019	0.020	0.037	0.021	0.069	0.370	0.982	0.998	0.989
PP25	0.047	0.031	0.045	0.020	0.034	0.053	0.030	0.014	0.026	0.041	0.024	0.018	0.018	0.042	0.021	0.083	0.416	0.968	0.994	0.981
PP26	-0.054	-0.055	-0.052	-0.050	-0.055	-0.054	-0.051	-0.053	-0.054	-0.057	-0.057	-0.051	-0.051	-0.057	-0.054	-0.058	-0.034	-0.017	-0.023	-0.015
PP27	0.029	0.008	0.028	0.008	0.021	0.026	0.032	0.009	0.026	0.021	0.012	0.010	0.027	0.023	0.013	0.050	0.170	0.023	0.050	0.046
AD01	0.104	0.074	0.106	0.056	0.071	0.106	0.114	0.017	0.068	0.079	0.056	0.070	0.036	0.091	0.102	0.206	-0.057	-0.034	-0.044	-0.045

AD02	0.403	0.539	0.434	0.497	0.495	0.510	0.489	0.419	0.470	0.529	0.447	0.537	0.414	0.569	0.561	0.556	0.087	-0.018	0.002	-0.003
AD03	0.349	0.441	0.372	0.400	0.407	0.431	0.418	0.327	0.385	0.433	0.364	0.438	0.334	0.471	0.467	0.502	0.060	-0.021	-0.006	-0.011
AD04	0.326	0.377	0.340	0.333	0.353	0.386	0.376	0.267	0.329	0.369	0.312	0.370	0.283	0.404	0.404	0.481	0.075	-0.016	0.000	-0.003
AD05	0.309	0.359	0.326	0.320	0.335	0.366	0.365	0.255	0.316	0.351	0.298	0.359	0.274	0.387	0.389	0.456	0.042	-0.021	-0.011	-0.014
AD06	0.445	0.597	0.478	0.564	0.559	0.563	0.543	0.482	0.525	0.583	0.504	0.598	0.474	0.623	0.622	0.591	0.102	-0.019	0.004	0.005
AD07	0.373	0.494	0.404	0.462	0.454	0.467	0.456	0.391	0.436	0.486	0.415	0.497	0.385	0.521	0.524	0.507	-0.022	-0.031	-0.033	-0.040
SD01	-0.104	-0.110	-0.098	-0.093	-0.106	-0.104	-0.100	-0.093	-0.100	-0.100	-0.102	-0.097	-0.093	-0.099	-0.103	-0.107	-0.052	-0.034	-0.044	-0.043
SD02	-0.122	-0.115	-0.115	-0.104	-0.106	-0.119	-0.103	-0.107	-0.116	-0.117	-0.118	-0.113	-0.106	-0.118	-0.116	-0.129	-0.061	-0.033	-0.046	-0.021
SD03	-0.085	-0.091	-0.080	-0.077	-0.086	-0.086	-0.082	-0.077	-0.083	-0.082	-0.084	-0.080	-0.077	-0.081	-0.085	-0.087	-0.043	-0.026	-0.032	-0.029
SD04	-0.097	-0.103	-0.091	-0.088	-0.099	-0.096	-0.094	-0.089	-0.095	-0.093	-0.096	-0.092	-0.088	-0.092	-0.097	-0.098	-0.042	-0.031	-0.037	-0.035
SD05	-0.144	-0.120	-0.136	-0.125	-0.119	-0.136	-0.123	-0.126	-0.136	-0.138	-0.137	-0.130	-0.125	-0.140	-0.132	-0.155	-0.079	-0.044	-0.060	-0.031
SD06	-0.110	-0.119	-0.104	-0.100	-0.114	-0.111	-0.107	-0.099	-0.107	-0.106	-0.108	-0.104	-0.099	-0.105	-0.111	-0.112	-0.039	-0.030	-0.043	-0.044
SD07	-0.079	-0.086	-0.075	-0.072	-0.083	-0.080	-0.078	-0.072	-0.078	-0.076	-0.079	-0.075	-0.072	-0.076	-0.080	-0.081	-0.040	-0.030	-0.035	-0.039
SD08	-0.096	-0.103	-0.090	-0.087	-0.099	-0.096	-0.093	-0.086	-0.093	-0.092	-0.094	-0.090	-0.086	-0.091	-0.096	-0.098	-0.050	-0.037	-0.044	-0.045
SD09	-0.103	-0.110	-0.097	-0.093	-0.106	-0.103	-0.099	-0.093	-0.100	-0.099	-0.101	-0.097	-0.092	-0.099	-0.103	-0.107	-0.053	-0.038	-0.046	-0.046
SD10	-0.095	-0.101	-0.089	-0.084	-0.095	-0.094	-0.089	-0.084	-0.092	-0.091	-0.092	-0.089	-0.084	-0.090	-0.094	-0.098	-0.057	-0.040	-0.045	-0.042

(Continued)

	PP05	PP06	PP07	PP08	PP09	PP10	PP11	PP12	PP13	PP14	PP15	PP16	PP17	PP18	PP19	PP20	PP21	PP22	PP23	PP24
PJ01																				
PJ02																				
PJ03																				
PJ04																				
PJ05																				
PJ06																				
PJ07																				
PJ08																				
PJ09																				
PJ10																				
PJ11																				
PJ12																				
PJ13																				
PJ14																				
PJ15																				
PJ16																				
PP01																				
PP02																				
PP03																				
PP04																				
PP05	1.000																			
PP06	0.968	1.000																		
PP07	0.998	0.954	1.000																	
PP08	0.983	0.994	0.973	1.000																
PP09	0.989	0.990	0.982	0.998	1.000															
PP10	0.925	0.988	0.905	0.970	0.963	1.000														

PP11	0.915	0.984	0.894	0.971	0.961	0.993	1.000													
PP12	0.958	0.997	0.941	0.985	0.980	0.990	0.983	1.000												
PP13	0.972	0.997	0.961	0.993	0.991	0.981	0.978	0.992	1.000											
PP14	0.994	0.980	0.990	0.993	0.997	0.947	0.944	0.969	0.985	1.000										
PP15	0.996	0.981	0.991	0.994	0.998	0.945	0.943	0.970	0.984	0.998	1.000									
PP16	0.997	0.960	0.997	0.976	0.982	0.913	0.902	0.950	0.966	0.991	0.991	1.000								
PP17	0.995	0.985	0.989	0.993	0.996	0.954	0.947	0.979	0.988	0.997	0.997	0.991	1.000							
PP18	0.994	0.977	0.989	0.982	0.986	0.943	0.928	0.974	0.980	0.989	0.990	0.992	0.996	1.000						
PP19	0.998	0.973	0.995	0.983	0.988	0.935	0.921	0.966	0.978	0.994	0.993	0.997	0.997	0.998	1.000					
PP20	0.993	0.970	0.990	0.974	0.980	0.932	0.914	0.965	0.974	0.986	0.986	0.993	0.992	0.999	0.998	1.000				
PP21	0.990	0.987	0.983	0.997	0.999	0.959	0.958	0.978	0.990	0.998	0.998	0.984	0.997	0.987	0.990	0.981	1.000			
PP22	0.972	0.899	0.980	0.936	0.949	0.834	0.833	0.877	0.911	0.966	0.964	0.975	0.954	0.949	0.963	0.949	0.953	1.000		
PP23	0.954	0.996	0.937	0.983	0.979	0.995	0.986	0.997	0.990	0.968	0.967	0.944	0.976	0.969	0.962	0.961	0.977	0.873	1.000	
PP24	0.984	0.995	0.974	0.998	0.996	0.970	0.969	0.989	0.995	0.992	0.993	0.978	0.994	0.987	0.986	0.981	0.995	0.932	0.985	1.000
PP25	0.972	0.998	0.958	0.997	0.993	0.981	0.982	0.992	0.996	0.984	0.986	0.962	0.986	0.976	0.974	0.967	0.991	0.912	0.990	0.997
PP26	-0.018	-0.025	-0.018	-0.022	-0.022	-0.031	-0.028	-0.023	-0.023	-0.019	-0.020	-0.014	-0.019	-0.019	-0.018	-0.018	-0.021	-0.008	-0.027	-0.020
PP27	0.028	0.059	0.020	0.039	0.034	0.075	0.065	0.071	0.054	0.027	0.028	0.025	0.039	0.046	0.035	0.045	0.032	-0.017	0.069	0.045
AD01	-0.038	-0.047	-0.035	-0.044	-0.043	-0.053	-0.051	-0.048	-0.044	-0.040	-0.039	-0.036	-0.040	-0.038	-0.038	-0.038	-0.042	-0.027	-0.050	-0.043
AD02	-0.020	0.010	-0.024	0.007	-0.002	0.017	0.033	0.007	0.016	-0.004	-0.007	-0.024	-0.002	-0.012	-0.015	-0.018	0.002	-0.030	0.012	0.009
AD03	-0.022	0.000	-0.025	-0.002	-0.009	0.005	0.018	-0.002	0.005	-0.009	-0.012	-0.025	-0.009	-0.016	-0.018	-0.021	-0.005	-0.028	0.001	0.000
AD04	-0.017	0.010	-0.021	0.007	-0.001	0.016	0.030	0.009	0.013	-0.003	-0.005	-0.021	-0.004	-0.012	-0.014	-0.017	0.001	-0.025	0.010	0.008
AD05	-0.022	-0.005	-0.024	-0.007	-0.012	-0.002	0.008	-0.007	-0.002	-0.013	-0.014	-0.025	-0.013	-0.019	-0.020	-0.022	-0.010	-0.026	-0.005	-0.005
AD06	-0.020	0.014	-0.025	0.011	0.000	0.023	0.040	0.012	0.019	-0.001	-0.005	-0.023	0.000	-0.012	-0.014	-0.019	0.005	-0.033	0.017	0.014
AD07	-0.034	-0.039	-0.031	-0.035	-0.037	-0.041	-0.038	-0.042	-0.031	-0.030	-0.034	-0.034	-0.028	-0.029	-0.030	-0.030	-0.031	-0.028	-0.036	-0.032
SD01	-0.037	-0.046	-0.036	-0.042	-0.042	-0.054	-0.048	-0.043	-0.041	-0.036	-0.038	-0.033	-0.036	-0.036	-0.036	-0.036	-0.039	-0.020	-0.048	-0.040
SD02	-0.037	-0.049	-0.037	-0.043	-0.046	-0.063	-0.056	-0.041	-0.045	-0.037	-0.041	-0.020	-0.038	-0.036	-0.035	-0.036	-0.043	-0.016	-0.055	-0.038
SD03	-0.026	-0.033	-0.025	-0.029	-0.029	-0.039	-0.035	-0.033	-0.031	-0.027	-0.026	-0.025	-0.028	-0.029	-0.028	-0.028	-0.029	-0.015	-0.036	-0.029
SD04	-0.032	-0.038	-0.032	-0.034	-0.034	-0.044	-0.038	-0.037	-0.035	-0.031	-0.031	-0.031	-0.032	-0.034	-0.033	-0.034	-0.033	-0.020	-0.041	-0.033
SD05	-0.050	-0.062	-0.049	-0.056	-0.059	-0.079	-0.072	-0.059	-0.059	-0.049	-0.052	-0.022	-0.050	-0.047	-0.046	-0.046	-0.056	-0.024	-0.071	-0.054
SD06	-0.038	-0.044	-0.039	-0.042	-0.043	-0.054	-0.046	-0.037	-0.036	-0.031	-0.039	-0.030	-0.032	-0.030	-0.031	-0.031	-0.038	-0.013	-0.046	-0.037
SD07	-0.030	-0.036	-0.030	-0.034	-0.033	-0.040	-0.038	-0.037	-0.033	-0.031	-0.030	-0.031	-0.031	-0.032	-0.032	-0.032	-0.033	-0.021	-0.039	-0.034
SD08	-0.037	-0.045	-0.036	-0.041	-0.040	-0.051	-0.046	-0.045	-0.043	-0.038	-0.037	-0.038	-0.039	-0.041	-0.040	-0.040	-0.040	-0.026	-0.048	-0.041
SD09	-0.039	-0.048	-0.039	-0.044	-0.043	-0.055	-0.050	-0.046	-0.044	-0.039	-0.040	-0.037	-0.040	-0.040	-0.039	-0.040	-0.042	-0.024	-0.050	-0.042
SD10	-0.037	-0.047	-0.036	-0.042	-0.041	-0.053	-0.050	-0.048	-0.047	-0.041	-0.038	-0.038	-0.043	-0.044	-0.042	-0.043	-0.043	-0.029	-0.051	-0.043

(Continued)

	PP25	PP26	PP27	AD01	AD02	AD03	AD04	AD05	AD06	AD07	SD01	SD02	SD03	SD04	SD05	SD06	SD07	SD08	SD09	SD10
PJ01																				
PJ02																				
PJ03																				
PJ04																				
PJ05																				
PJ06																				
PJ07																				
PJ08																				

PJ09															
PJ10															
PJ11															
PJ12															
PJ13															
PJ14															
PJ15															
PJ16															
PP01															
PP02															
PP03															
PP04															
PP05															
PP06															
PP07															
PP08															
PP09															
PP10															
PP11															
PP12															
PP13															
PP14															
PP15															
PP16															
PP17															
PP18															
PP19															
PP20															
PP21															
PP22															
PP23															
PP24															
PP25	1.000														
PP26	-0.024	1.000													
PP27	0.050	-0.022	1.000												
AD01	-0.045	-0.032	0.044	1.000											
AD02	0.010	-0.037	0.103	0.621	1.000										
AD03	0.001	-0.034	0.090	0.703	0.977	1.000									
AD04	0.012	-0.032	-0.007	0.750	0.928	0.974	1.000								
AD05	-0.004	-0.032	-0.011	0.760	0.918	0.973	0.995	1.000							
AD06	0.014	-0.038	0.010	0.563	0.978	0.945	0.904	0.891	1.000						
AD07	-0.041	-0.036	0.032	0.655	0.980	0.973	0.935	0.932	0.959	1.000					
SD01	-0.043	0.022	-0.037	-0.057	-0.063	-0.058	-0.053	-0.055	-0.068	-0.063	1.000				
SD02	-0.049	0.013	-0.040	-0.061	-0.071	-0.065	-0.061	-0.062	-0.067	-0.069	0.818	1.000			
SD03	-0.031	0.004	-0.032	-0.053	-0.057	-0.054	-0.049	-0.050	-0.061	-0.058	0.963	0.863	1.000		
SD04	-0.034	-0.002	-0.036	-0.059	-0.062	-0.058	-0.052	-0.055	-0.066	-0.065	0.836	0.919	0.879	1.000	
SD05	-0.062	0.036	-0.050	-0.074	-0.084	-0.078	-0.072	-0.072	-0.084	-0.084	0.837	0.803	0.796	0.763	1.000

SD06	-0.043	0.023	-0.033	-0.050	-0.053	-0.047	-0.044	-0.048	-0.060	-0.055	0.968	0.760	0.888	0.746	0.815	1.000				
SD07	-0.033	-0.003	-0.030	-0.048	-0.052	-0.049	-0.044	-0.046	-0.057	-0.053	0.720	0.868	0.797	0.969	0.666	0.607	1.000			
SD08	-0.042	-0.003	-0.037	-0.061	-0.065	-0.061	-0.055	-0.057	-0.070	-0.065	0.906	0.899	0.929	0.982	0.792	0.822	0.925	1.000		
SD09	-0.045	0.005	-0.038	-0.060	-0.065	-0.061	-0.055	-0.057	-0.070	-0.065	0.914	0.908	0.917	0.973	0.815	0.849	0.909	0.982	1.000	
SD10	-0.044	-0.018	-0.038	-0.061	-0.069	-0.066	-0.059	-0.060	-0.071	-0.067	0.415	0.401	0.457	0.407	0.330	0.363	0.388	0.425	0.408	1.000
